# Acceptability, values, and preferences of older people for chronic low back pain management; a qualitative evidence synthesis

**DOI:** 10.1186/s12877-023-04608-4

**Published:** 2024-01-05

**Authors:** Heather Ames, Christine Hillestad Hestevik, Andrew M. Briggs

**Affiliations:** 1 The Norwegian Institute of Public Health, PO Box 222, 0213 Oslo, Skøyen Norway; 2 Ageing and Health Unit, Department of Maternal, Newborn, Child & Adolescent Health and Ageing, World Health Organization, Avenue Appia 20, 1211 Geneva, Switzerland; 3 Faculty of Health Sciences, Curtin University, PO Box U1987, Perth, 6845 Western Australia

**Keywords:** Chronic, Low back pain, Interventions, Qualitative evidence synthesis, Older adults

## Abstract

**Background:**

Chronic primary low back pain (CPLBP) and other musculoskeletal conditions represent a sizable attribution to the global burden of disability, with rates greatest in older age. There are multiple and varied interventions for CPLBP, delivered by a wide range of health and care workers. However, it is not known if these are acceptable to or align with the values and preferences of care recipients. The objective of this synthesis was to understand the key factors influencing the acceptability of, and values and preferences for, interventions/care for CPLBP from the perspective of people over 60 and their caregivers.

**Methods:**

We searched MEDLINE, CINAHL and OpenAlex, for eligible studies from inception until April 2022. We included studies that used qualitative methods for data collection and analysis; explored the perceptions and experiences of older people and their caregivers about interventions to treat CPLBP; from any setting globally. We conducted a best fit framework synthesis using a framework developed specifically for this review. We assessed our certainty in the findings using GRADE-CERQual.

**Results:**

All 22 included studies represented older people’s experiences and had representation across a range of geographies and economic contexts. No studies were identified on caregivers. Older people living with CPLBP express values and preferences for their care that relate to therapeutic encounters and the importance of therapeutic alliance, irrespective of the type of treatment, choice of intervention, and intervention delivery modalities. Older people with CPLBP value therapeutic encounters that validate, legitimise, and respect their pain experience, consider their context holistically, prioritise their needs and preferences, adopt a person-centred and tailored approach to care, and are supported by interprofessional communication. Older people valued care that provided benefit to them, included interventions beyond analgesic medicines alone and was financially and geographically accessible.

**Conclusions:**

These findings provide critical context to the implementation of clinical guidelines into practice, particularly related to how care providers interact with older people and how components of care are delivered, their location and their cost. Further research is needed focusing on low- and middle-income settings, vulnerable populations, and caregivers.

**Supplementary Information:**

The online version contains supplementary material available at 10.1186/s12877-023-04608-4.

## Background

Low back pain (LBP) and other musculoskeletal conditions represent a sizable contribution to the global burden of disability [1–5]. While the prevalence and impact of LBP are relevant across the life-course, global estimates for prevalence and disability show rates to be greatest in older age. The high prevalence of LBP in older people accounts for frequent care seeking for LBP [6], particularly among older adults experiencing recurrent LBP [7]. The number of older people experiencing and seeking care for LBP is expected to increase due to population ageing and an increasing prevalence of risk factors for noncommunicable diseases [8]. Despite this, intervention trials and clinical guidelines for LBP disproportionately underrepresent older people [9, 10], potentially leaving an important knowledge gap for optimal care of LBP in older people.

Clinical management of LBP is characterized by multiple and varied interventions, delivered by a wide range of health and care workers [11–20]. In many contexts the interventions delivered may not be aligned with best evidence leading to unwarranted care variation and potential harm. Further, interventions may not be aligned with the values, preferences and acceptability attitudes among care recipients (and/or their carers), substantiating the need for global guidelines in this area [21]. Importantly, values and preferences of older people likely differ to younger adults. From the perspective of healthy ageing, carers are an essential workforce for supporting functional ability in older people and enabling ageing in place. The perspectives of carers are therefore critical to ensure care planning and delivery for any health condition experienced by an older people is feasible and acceptable and does not negatively impact on the quality of life of the carer [22, 23] . For example, recent work has also identified the need to sample perspectives of carers related to delivery of care for people living with chronic pain [24].

In response to this context and the priority to support healthy ageing, the World Health Organization (WHO) initiated the development of standard clinical guideline for the non-surgical management of chronic primary LBP (CPLBP) in adults, including older people, in primary and community care settings in 2020 [21]. The guidelines were published in December 2023 [25].

This qualitative evidence synthesis was commissioned in parallel to several systematic reviews of evidence of benefits and harms of prioritized interventions for the Guideline, synthesized from randomized controlled trials (RCTs) [26–44]. These interventions were broad in scope, intensity and setting for delivery (reflected in the inclusion criteria for this synthesis). The aim of all the interventions is to improve health and wellbeing outcomes for people living with CPLBP. However, it is important to explore how this broad variation in interventions is perceived and experienced by older people with CPLBP and/or their caregivers (formal or informal, family members). Are some interventions more accepted than others? Are there differences between the interventions and/or access to them related to equity (gender, culture, place of residence, socio economic status) or setting (geographic or health care setting)? These important context questions can only be comprehensively answered using qualitative research methods. These contextual data are intended to support the development of the WHO guideline and complement additional perspectives brought to the development process by other stakeholders involved in the guideline development, consistent with WHO guideline development methods [45].

It is important to consider people’s preferences around interventions when formulating and implementing clinical management guidelines. In this paper we use the concept of person-centred care, in order to encompass a broader perspective than those related to being a patient. We have adopted the definition of person-centred care that is used in the WHO Guideline, that is “Person-centred care means eliciting an individual’s values, preferences and priorities: once expressed, they should guide all aspects of that person’s health care, supporting their personalized health and life goals” [46, 47].

An intervention may be proven effective but if it is not accepted by people living with the condition (and/or their carers) or they feel it causes burden or harm, it will not be adopted. An important step in a WHO guidelines development process is to consider what people living with CPLBP and their caregivers find acceptable? Feasible? Valued? [45] For example, there is a need to understand preferences and perspectives concerning accessibility, availability, affordability, perceived quality, burden [time, distance, frequency of visits], stigma, duration of therapeutic effect, person/patient’s role (passive or active role), immediacy of treatment effect, configuration of the care team– single practitioner or team approach, influence on comorbid health conditions, and symptoms related to the treatment. Some of these dimensions of value, preference and acceptability have been identified as previously as important to decision-making around treatments among older adults with osteoarthritis [48]. To date there has been some research conducted that considers people’s preferences for treatment for CPLBP [49–55]. However, to our knowledge, there has been no synthesis of primary qualitative research exploring the key factors influencing the implementation, uptake, and experience of interventions designed to manage CPLBP from the perspective of people aged over 60 and their caregivers.

The objective of this qualitative evidence synthesis (QES) was to understand the key factors influencing the acceptability of, and values and preferences/perspectives for, interventions/care for CPLBP from the perspective of people over 60 and their caregivers. The purpose of the QES was to inform the development of the WHO guideline [25].

## Methods

This QES followed the best practice as described by the Cochrane collaboration in their handbook [56, 57]. The protocol was registered on PROSPERO at inception (https://www.crd.york.ac.uk/prospero/display_record.php?RecordID=328469).

We included primary studies with qualitative study designs. We included mixed-methods studies when it was possible to extract the data that were collected and analysed using qualitative methods. The inclusion criteria are described in Table 1.

**Table 1:** Inclusion criteria

**Perspective**	Adults aged 60 years and over with CPLBP and/or their caregivers (formal or informal, family members), including studies where the mean age of participants is 60 or over in high-income settings. For cohorts sampled in low- or middle-income countries (LMIC) we lowered the age threshold to the second half of life, based on median life expectancy for the country, as defined by WHO. We also applied this criterion to vulnerable population groups within a high-income country, based on median life expectancy of the that vulnerable group (e.g., indigenous populations or other ethnic minority groups).
**Setting**	Primary or community care, residential aged care/supportive care facility, or any community setting
**Phenomenon (topic) of interest (equivalent to the five intervention [PICO] classes)**	The acceptability of, and values and preferences for, interventions/care for CPLBP Interventions of interest include: • Medicines • Physical therapies • Psychological therapies • Education • Multi-component interventions We applied the same operational definition as adopted by the WHO Guideline, consistent with ICD-11 and the IASP definition of chronic primary pain (low back pain). Specifically, CPLBP was defined as “pain that persists or recurs for longer than three months and is associated with symptoms that cannot be better accounted for by another diagnosis, such as a structural lesion or a disease process. No criteria were applied relating to an experience of emotional distress or functional disability.” [58]
**Time/Timing**	CPLBP experienced continuously or recurrently for more than 3 months
**Findings**	Older adult participant’s perspectives, experiences or insights regarding to values, preferences, cost/resources, acceptability and equity [45]

We searched in two databases (MEDLINE and CINAHL powered by Ovid) (April 28, 2022) and supplemented the search with a search in an open-source dataset, OpenAlex [59, 60] (May 3, 2022) through the EPPI-Reviewer platform [59]. We also screened the references of the included studies. Finally, we asked members of the WHO Guideline Development Group to recommend any relevant research they were aware of.

To maximise efficiency of the study selection process, we used the machine learning function “priority screening” in the systematic review software EPPI-reviewer [61].

Two review authors (HA and CHH) independently assessed eligibility of the titles and abstracts. We retrieved the full text of all the papers identified as potentially relevant. Two authors (HA and CHH) then assessed the eligibility of these papers independently. Discrepancies in decisions were resolved by discussion among the authors.

Data extraction was performed using a data extraction form designed specifically for this review. One author performed the data extraction and a second author checked for accuracy against the source paper and any discordances were resolved through consensus discussion. We extracted the following information from the studies; author, year of publication, geographic setting, description of context, data collection methods (sampling, collection, and analysis), description of participants covering the aspects named in the inclusion table (see Table 1) and if ethics approval was given for the study.

We assessed the methodological limitations of the included studies using a list of domains iteratively developed by the Cochrane EPOC group [62–65]. We did not exclude studies based on our assessment of methodological limitations but used the information about methodological limitations to assess our confidence in the review findings.

We analysed the data by conducting a best fit framework synthesis [66–69]. Best fit framework synthesis is a qualitative synthesis method that blends deductive and inductive synthesis and analysis processes. As part of the synthesis method, review authors identify a conceptual framework that fits at least 50% of the data. After data extraction, data that does not fit within the framework is further analysed in order to develop a new framework that includes all of the data. We used the themes identified in the scoping review on older adults’ perceptions and experiences of integrated care by Lawless et al. [70], a conceptual framework from Chua et al. on choosing interventions for hip or knee osteoarthritis [48] as well as the PROGRESS Plus framework that addresses issues related to equity [71] to generate an a priori theoretical framework. We chose these frameworks as they were relevant to the topic we were exploring and expected to cover at least 50% of the data. The PROGRESS+ framework [71] was added to address the specific needs of the WHO guidelines process around equity, gender and human rights. HA moved the extracted data into the framework and CHH checked the data. We then analysed the data within each framework section and developed our findings. Relevant data that did not fit into the framework were analysed thematically. We used a thematic analysis approach as described by Miles and Huberman [72] as referred to in Carroll 2013 [66] in their paper on best fit framework synthesis. New themes were generated based on our interpretation of the evidence and constant comparison of the new themes across the included studies. In accordance with best fit framework synthesis methods, we inductively expanded the a priori framework to include a section on person-centred care and communication to reflect the breadth of all our findings.

Findings were then organized according to the domains defined in the WHO Handbook for Guideline Development that inform the determination of a recommendation, derived from qualitative evidence, including values and preferences, resource implications, equity and human rights, acceptability and feasibility (See Table 2).

**Table 2:** Descriptions of Evidence to Decision (EtD) factors that determine the direction and strength of a recommendation in WHO guidelines [45] (page 124)

EtD factor	How the factor influences the direction and strength of a recommendation
Values and preferences	This describes the relative importance assigned to health outcomes by those affected by them; how such importance varies within and across populations; and whether this importance or variability is surrounded by uncertainty. The less uncertainty or variability there is about the values and preferences of people experiencing the critical or important outcomes, the greater the likelihood of a strong recommendation.
Resource implications	This pertains to how resource-intense an intervention is, whether it is cost–effective and whether it offers any incremental benefit. The more advantageous or clearly disadvantageous the resource implications are, the greater the likelihood of a strong recommendation either for or against the intervention.
Equity and human rights	The greater the likelihood that the intervention will reduce inequities, improve equity, or contribute to the realization of one or several human rights as defined under the international legal framework, the greater the likelihood of a strong recommendation.
Acceptability	The greater the acceptability of an option to all or most stakeholders, the greater the likelihood of a strong recommendation.
Feasibility	The greater the feasibility of an option from the standpoint of all or most stakeholders, the greater the likelihood of a strong recommendation. Feasibility overlaps with values and preferences, resource considerations, existing infrastructures, equity, cultural norms, legal frameworks, and many other considerations.

Finally, we assessed our confidence in the findings using GRADE-CERQual [73]. We present detailed descriptions of our confidence assessment in Evidence Profile(s) [74].

In each section we present the summary of findings table and a summary of the main points discussed in the findings. For specific findings and our confidence in them please refer to Tables 4-9 (Summary of Qualitative Evidence Tables).

### Review author reflexivity

Neither Heather Ames (HA), Christine Hillestad Hestevik (CHH) or Andrew Briggs (AMB) have reached the age of 60, so we do not understand the lived experience of being an older adult. HA is a previous elite athlete who has experience with chronic musculoskeletal pain and interventions due to injury and AMB has experience of chronic musculoskeletal pain. Both HA and AMB’s parents are over 60, have experienced chronic pain and have discussed their treatments with them. All authors support an evidence-based medicine approach to care. AMB is a clinician, researcher, and health systems professional in the field of chronic musculoskeletal pain. CHH does not have personal experience with chronic musculoskeletal pain or treatment interventions. She did her PhD on healthcare provided to older people from the perspectives of older persons, their family caregivers and healthcare professionals and has some experience with older persons experiences with encounters when in need of healthcare. These prior experiences, particularly a lived experience of chronic musculoskeletal pain, lead us to believe in the difficulties older people are facing. It also felt like the topics that were being raised were familiar from the perspective of personal and research experience.

## Findings

**Fig. 1:**
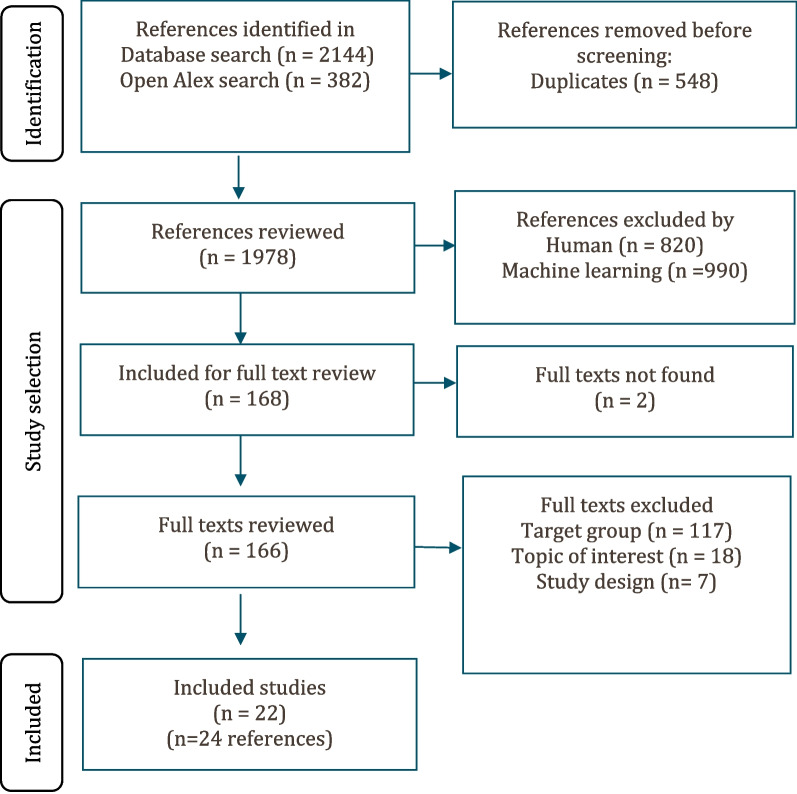
Study selection flowchart

From a yield of 1878 unique citations, 22 studies were included in this review, reflected in 24 reports. See Fig. 1 for the study selection process. For a description of the included studies see Table 3.

**Table 3:** Characteristics of included studies

Author (year)	Country (HIC or LMIC/ vulnerable group)	Population characteristics	Setting	Phenomenon (topic) of interest	Data collection methods and analytic approach
Allvin (2019) [75]	Sweden (HIC)	*N* = 9, aged 39–74 years, median age 66 years, 55% women	Participants were admitted to the hospital by a referral from primary care doctors or through the emergency department	Experience encounters with health care.	Individual semi-structured interviews, qualitative content analysis
Bonfim (2021) [76]	Brazil (LMIC)	*N* = 70, mean age 60.7 (SD 13), 83% women	Community dwelling adults who visited Physical therapy outpatient services in the city of Rio de Janeiro	Perceptions regarding the influence of clinical diagnosis on pain, beliefs, and daily life activities.	Individual semi-structured interviews, discourse content analysis
Cooper (2017) [77]	Scotland (HIC)	*N* = 36, aged 65 years and above, 75% women	Community dwelling older adults who were discharged from physiotherapy 3 to 6 months before the study	Peer-mentoring	Individual semi-structured interviews, framework analysis
Cummings (2017) [78]	USA (HIC)	*N* = 20, aged 29–78 years, with 10 participants aged over 60, 80% women	Community dwelling adults	Patient narratives about the experience of chronic back pain	Semi-structured interviews, inductive thematic analysis
Dima (2013) [79]	England, (HIC)	*N* = 75, aged 29–85 years old (median age 62 years), 64% women	Adults who had recently consulted their family doctor or CAM practitioner because of LBP	LBP treatments (medication, exercise, manual therapy, acupuncture, combined psychological and physical treatment programs, or spinal surgery), to other treatments, or to clinical management of LBP)	Focus group interviews, thematic analysis
Hay (2020) [80]	Canada (HIC)	*N* = 10, aged 66–97, 70% women	Community-dwelling	Regular exercise	Individual interviews, hermeneutic phenomenology
Igwesi Chidobe (2019 and 2020) [81, 82]	Nigeria (LMIC)	*N* = 22, mean age 53. 9 (SD 14.1), 76.9% women	Community-dwelling	The Good Back program, a six-week group self-management program that incorporates individual exercise sessions with discussion sessions; administered once weekly	Semi structured interviews, inductive content analysis
Igwesi-Chidobe (2017) [83]	Nigeria (LMIC)	*N* = 30, aged 30–69, 50% women	Community-dwelling peasant farmers	Experiences of people living with non-specific chronic low back pain (CLBP) in a rural Nigerian community	Semi-structured individual interviews, thematic analyses using the framework approach
Kirby (2014) [84]	Australia (HIC)	*N* = 50, aged 60–65 years, 100% women	The project was conducted as part of the Australian Longitudinal Study on Women’s Health	The influences on back pain sufferers’ decision-making about treatment seeking with practitioners for their most recent episode of back pain.	Telephone based semi structured interviews, systematic thematic content analysis of the data employing a framework approach
Kuss (2016) [85]	Germany (HIC)	*N* = 16, aged 73.9 (SD 5.9), 81.3% women	Patients were referred by their general practitioners, who screened for inclusion criteria in the program	The GA program, twelve 45-minute sessions over a 9-week period.	Individual interviews and videos of the sessions were analysed with content analysis
Lee (2020) [54]	USA (HIC)	*N* = 18, aged 65 or above, 61% women	Community dwelling	A 36-week t’ai chi intervention beginning with twice weekly classes for 12 weeks, weekly classes for 6 weeks, biweekly classes for 6 weeks, and monthly classes for 12 weeks	Focus group interviews, grounded theory
Leonhardt (2017) [86]	Germany (HIC)	*N* = 16, mean age 72.6 (SD 4.7), 62,5% women	A primary care setting	A cognitive-behavioural exposure-based physical therapy program	Semi structured, content analysis
Lilje (2017) [87]	Sweden (HIC)	*N* = 15, aged, mean age 76,7 (range 67–86), 53,3% women	Community dwelling	Mobile phone text messaging as reminders of home exercises after specialized manual therapy	Semi-structured interview, systematic text condensation
Lin (2013 and 2014) [88, 89]	Australia (HIC/ Aboriginal population)	*N* = 32, aged 26–72 years, 34.4% women	Community dwelling	Low back pain beliefs of Aboriginal Australians	In-depth interviews informed by clinical ethnography and cultural security. Informed by clinical ethnography and cultural security.
Luiggi-Hernandez (2018) [90]	USA (HIC)	*N* = 25, age mean 76.6 (SD 7.1), 60% women	Community dwelling	Eight-week mindfulness program	Focus group interviews/ thematic analysis
Lyons (2013) [91]	USA (HIC)	*N* = 48, mean age (75.2 (SD 8.0) years, 79% women	Community dwelling	Preferences of older adults for LBP co-management by medical doctors and Doctor of Chiropractic	Focus group interviews, content analysis
MacKichan (2013) [92]	UK (HIC)	*N* = 23, aged 38–83 years, 47,8% women	Participants were identified through a postal survey of a stratified sample of 3060 adults who were identified at random through patient records at three GP practices	Patients’ experience of self-care for long-term back pain and their views on provision of support for self-care.	In depth interviews, constant comparative method
Makris (2015) [93]	USA (HIC)	*N* = 93, median age 83, 68% Women, the years,	Community dwelling	Beliefs and perspectives regarding care-seeking for restricting back pain	23 semi-structured interviews and 16 focus groups, an inductive text driven approach to qualitative thematic analysis
Morone (2008) [94]	USA (HIC)	*N* = 27, mean age 74.3 (SD 5.3), 52% women	Community dwelling	Clinical trial of an 8-week mindfulness meditation program	Content analysis of meditation diaries written by older adults
Rodriguez 2019 [95]	Chile (HIC)	*N* = 10, aged 67–79, 80% women	Community dwelling	Experience of chronic pain produced by spinal deformity	Semi-structured interviews, grounded theory
Stensland (2021) [53]	USA (HIC)	*N* = 21, mean age 71 years (SD 5.5), 62% women	Community dwelling	Experiences of pain management approaches	Semi-structured interviews/ phenomenology
Teh (2009) [96]	USA (HIC)	*N* = 15, aged 63–86 years, 67% women	Patients had been referred to a university-based pain clinic by their primary care providers and had previously participated in a study of older adults with chronic pain	Experiences of seeking treatment for chronic pain, with respect to patient-directed care and the patient–provider relationship	In-depth interviews, grounded theory

*HIC* High income country, *LMIC* low- and middle-income countries

The included studies were conducted in the United States (*n* = 8) [53, 54, 78, 90, 91, 93, 94, 96], United Kingdom (*n* = 3) [77, 79, 92], Germany (*n* = 2) [85, 86], Sweden (*n* = 2) [75, 87], Australia (*n* = 2) [84, 88, 89], Canada (*n* = 1) [80], Chile (*n* = 1) [95], Brazil (*n* = 1) [76], and Nigeria (*n* = 2) [81–83]. One study focused on Aboriginal Australians, a vulnerable population [88, 89]. In 14 of the studies all participants were aged 60 or older [53, 54, 77, 80, 84–87, 90, 91, 93–96]. In five, the mean or median age of the participants were 60 or older [75, 76, 78, 79, 92]. Three studies were included under the inclusion criteria for age for a low or middle-income country or identified vulnerable population [75, 81–83, 88, 89]. In these studies, the age of the participants ranged from 26 to 72 years, but we only used disaggregated results from participants aged 40 or above.

In 16 of the studies, the participants were community-dwelling older adults [53, 54, 76–78, 80–83, 87–91, 93–96]. Three of the studies were conducted in a primary health care setting but the residence of the participants was not discussed [75, 85, 86]. In three studies, the setting was unclear so we could not define the residence of the participants [79, 84, 92]. Nine of the studies were nested in a trial or a larger feasibility study [53, 54, 84, 86, 90–94].

We did not identify any studies that explored the perceptions or experiences of caregivers (formal or informal, family members).

## Acceptability, values, and preferences

Since there was a large overlap in evidence related to values and preferences and acceptability, the findings are presented pooled. Values and preferences extended to interactions with health care providers, interventions for CPLBP and the modes of care delivery for CPLBP. Sixteen studies from 11 countries contributed to these findings (USA, Germany, Australia, United Kingdom, England, Scotland, Canada, Nigeria, Sweden, Brazil, and Chile). Participants in nine studies were all over 60 [54, 77, 80, 85, 87, 91, 93, 95, 96]. Four studies had participants with a mean or average age of 60 or older [75, 76, 79, 92] and four studies were from LMICs or vulnerable populations [76, 81–83, 88, 89] of which three were included based on a lowered age threshold [81–83, 88, 89]. In 13 of the studies most of the participants were women (53–83%) [54, 75–77, 79–82, 85, 87, 91, 93, 95, 96]. In two studies [83, 92] there was an equal distribution of men and women. In one study most participants were men (52–66% men) [88, 89].

### Interactions with health care providers

Most participants wanted their health care providers to collaborate and work together to provide holistic care for their CPLBP. There was a preference among participants for providers who were respectful, caring, person-centred, collaborative, open to discussing treatment options and provided individualized care. They preferred health care providers who recognized them and their pain as individual and unique. This type of care allowed them to feel safe and feel they had meaningful relationships. When this was lacking, they could feel frustrated, vulnerable and experience a sense of aloneness (high confidence) [75, 79, 83, 88, 89, 91, 93, 95, 96].

Participants generally emphasized the care should be person-centred and provide continuity. They also identified a preference for a collaborative communication style which meant involving the older person in discussions about diagnosis and treatment options and viewing them as the expert on their own body (low confidence) [77, 79, 80, 88, 91].

Participants wanted collaboration and communication across their care teams to ensure co-ordinated care delivery and avoid duplication in care or diagnostics (moderate confidence) [75, 88, 91]. Some participants felt that they often received conflicting advice or information from health care providers. Participants valued receiving a diagnosis as this influenced their treatment decisions. The way the diagnosis was communicated could also shape their beliefs and responses to pain (moderate confidence) [76, 79, 81, 83, 85, 89, 91, 92, 95]. Some participants expressed dissatisfaction with health care providers for being unwilling to discuss treatment options other than medication (low confidence) [75, 93, 96]. The summary of findings is presented in Table 4.

**Table 4:** Summary of qualitative findings table: Acceptability, values, and preferences for interactions with health care workers in older people

#	Summarised review finding	GRADE-CERQual Assessment of confidence	Explanation of GRADE-CERQual Assessment	References
1	Many participants preferred providers who treated them with respect, cared for their individual needs and recognized the patient as the expert. They became frustrated when they were not taken seriously, disbelieved, were not treated as a person, experienced a lack of dialogue or clear, specific information or other conditions were prioritized over their CPLBP. This could be a deterrent to future treatment or care seeking. Many participants valued a health care provider who understood, listened, and remembered them. If this was lacking participants could feel not cared about, vulnerable, or alone with their pain. They wanted meaningful relationships with their providers who could sympathize, understand, and see them as a whole person. This understanding could help to legitimize and validate their pain experience.	High confidence	Minor concerns regarding methodological limitations, No/Very minor concerns regarding coherence, No/Very minor concerns regarding adequacy, and No/Very minor concerns regarding relevance	Lyons Kevin J et al. 2013; Makris Una E et al. 2015; Allvin R et al. 2019; Dima A et al. 2013; Lin I B et al. 2013; Lin I et al. 2014; Igwesi-Chidobe C N et al. 2017; Rodriguez I et al. 2019; Teh Carrie F et al. 2009;
2	Participants generally emphasized that there was a need for individualized care and guidance (for example how to perform an exercise) across the different interventions, whether health-professional or peer delivered. Care should be person-centred and provide continuity. Supervision/professional guidance allowed older people to feel safe. Several participants reported the importance of having an instructor for exercise or group classes who was personable, knowledgeable, and interactive and gave each participant individual attention. There was a preference for a collaborative communication style.	Low confidence	Minor concerns regarding methodological limitations, Moderate concerns regarding coherence, No/Very minor concerns regarding adequacy, and Minor concerns regarding relevance	Lyons Kevin J et al. 2013; Cooper K et al. 2017; Hay M E & Connelly D M 2020; Dima A et al. 2013; Lin I et al. 2014; Kuss K et al. 2016; Igwesi-Chidobe C N et al. 2020; Lilje S C et al. 2017; Igwesi-Chidobe C N et al. 2019; Teh Carrie F et al. 2009; Lee T L et al. 2020;
3	Participants generally agreed that there should be collaboration and communication across care teams/ different healthcare providers to ensure adequate treatment and to avoid duplication in testing, treatments and ensure consistency of recommendations and information across providers. Some participants had experienced no or a lack of communication between their health care providers (within or across specialties) concerning their diagnosis and plan of care or had received conflicting advice.	Moderate confidence	No/Very minor concerns regarding methodological limitations, No/Very minor concerns regarding coherence, Minor concerns regarding adequacy, and Moderate concerns regarding relevance	Lyons Kevin J et al. 2013; Allvin R et al. 2019; Lin I et al. 2014;
4	It was important for participants to receive a diagnosis. This influenced their treatment decisions, self-management decisions and how they viewed themselves and their prognosis. The way a diagnosis is communicated can shape the patient’s beliefs and response to their CPLBP. Inadequate or incorrect information influenced participants to view their diagnosis as threatening and as a reason for changing their daily activities. Participants preferred clear, honest and adequate information about diagnosis and prognosis and treatment.	Moderate confidence	Minor concerns regarding methodological limitations, Moderate concerns regarding coherence, No/Very minor concerns regarding adequacy, and No/Very minor concerns regarding relevance	Lyons Kevin J et al. 2013; Dima A et al. 2013; Lin I B et al. 2013; Bonfim I D. S et al. 2021; Lin I et al. 2014; Igwesi-Chidobe C N et al. 2017; MacKichan F et al. 2013; Rodriguez I et al. 2019; Kuss K et al. 2016; Igwesi-Chidobe C N et al. 2019;
5	Some participants expressed dissatisfaction with health care providers for being unwilling to discuss treatment options other than medication. Some of these participants had different priorities than their health care providers and felt that their providers were not meeting their needs by only providing medication and giving inadequate or conflicting information.	Low confidence	Minor concerns regarding methodological limitations, Minor concerns regarding coherence, Moderate concerns regarding adequacy, and Moderate concerns regarding relevance	Makris Una E et al. 2015; Allvin R et al. 2019; Teh Carrie F et al. 2009;

### Values and preferences for CPLBP interventions in older people

Participants had clear values and preferences for how they chose a specific treatment for CPLBP. A choice of treatment could be influenced by previous experiences. Participants valued treatments that they viewed as effective, beneficial, and credible and fit them as individuals (high confidence) [53, 54, 79–82, 84–87, 93, 95, 96].

Most participants used and valued medication for its ability to provide short-term pain relief. However, many participants did not like the side effects associated with medications or the way the medication(s) made them feel (moderate confidence) [53, 78, 79, 91, 93, 96]. Many also feared addiction, especially in relation to opioid analgesics. In some cases, participants adjusted or stopped medication without consulting their health care provider because of fears of adverse events (moderate confidence) [53, 79, 91, 96, 97].

Mindfulness and meditation encouraged participants to examine, assess, understand, and accept their pain rather than avoid it. Participants were able to use mindfulness and meditation for pain management and coping to varying degrees (moderate confidence) [54, 90, 94]. The summary of the findings is presented in Table 5.

**Table 5:** Summary of qualitative findings table: Acceptability, values, and preferences for CPLBP interventions in older people

#	Summarised review finding	GRADE-CERQual Assessment of confidence	Explanation of GRADE-CERQual Assessment	References
6	Patients had clear preferences and values for how they chose a specific treatment for CPLBP, which could be influenced by previous experiences. They valued and preferred treatments that they experienced as effective, beneficial and credible. In some cases, they also valued treatments that fit them as individuals (personally enjoyable, positive impact, meaningful, involved social engagement).	High confidence	No/Very minor concerns regarding methodological limitations, Minor concerns regarding coherence, No/Very minor concerns regarding adequacy, and No/Very minor concerns regarding relevance	Makris Una E et al. 2015; Stensland M 2021; Hay M E & Connelly D M 2020; Dima A et al. 2013; Rodriguez I et al. 2019; Kuss K et al. 2016; Igwesi-Chidobe C N et al. 2020; Lilje S C et al. 2017; Igwesi-Chidobe C N et al. 2019; Teh Carrie F et al. 2009; Lee T L et al. 2020; Kirby E R et al. 2014; Leonhardt Corinna et al. 2017;
7	Many participants experienced that medication was often the only intervention that made a difference to the severity of their pain. However, they were apprehensive of, or dissatisfied with, medication for a number of reasons, often viewing it as a quick fix, temporary relief, or that it just masked the pain. Many participants were apprehensive of taking too many medications, the side effects, risk of addiction or did not like how the medications made them feel. Some avoided taking medication all together, filling prescriptions or adjusted medication themselves because of perceived risks of adverse events.	Moderate confidence	Minor concerns regarding methodological limitations, No/Very minor concerns regarding coherence, No/Very minor concerns regarding adequacy, and Moderate concerns regarding relevance	Lyons Kevin J et al. 2013; Makris Una E et al. 2015; Stensland M 2021; Cummings E C et al. 2017; Dima A et al. 2013; Teh Carrie F et al. 2009;
8	Many participants expressed a fear of addiction to medication, especially to opioid analgesics. This led them to not fill prescriptions, to adjust the dosage or stop taking the medication often without consulting their health care provider. In one case, the fear of addiction came from the health care provider who then refused to give the prescription requested.	Moderate confidence	Minor concerns regarding methodological limitations, No/Very minor concerns regarding coherence, Minor concerns regarding adequacy, and Moderate concerns regarding relevance	Lyons Kevin J et al. 2013; Makris Una E et al. 2015; Stensland M 2021; Dima A et al. 2013; Teh Carrie F et al. 2009;
9	Mindfulness and meditation encouraged participants to examine, assess, understand and accept their pain rather than avoid it. It allowed some participants to increase their body awareness in relation to, for example, breathing, posture, cognition and pain, resulting in a perceived decrease in the significance or power of their pain experience. Others were able to use mindfulness and meditation for pain management and coping to varying degrees.	Moderate confidence	Minor concerns regarding methodological limitations, No/Very minor concerns regarding coherence, No/Very minor concerns regarding adequacy, and Moderate concerns regarding relevance	Luiggi-Hernandez J G et al. 2018; Morone N E et al. 2008; Lee T L et al. 2020;

### Format of interventions and educational materials for CPLBP in older people

Participants discussed their experiences with, and views of, organized and unorganized physical therapies and activities. Specific physical interventions were rarely mentioned. For many participants, physical activity was an important aspect of coping with their CPLBP. Many participants preferred a group format for physical exercises as it facilitated social support, collaboration and encouraged increased attendance (moderate confidence) [54, 79–82, 85]. Some participants also expressed preferences for educational material for physical interventions which had drawings and descriptions of the exercises. This made them more comprehensible, easier to follow and helpful for present and future reference (low confidence) [79, 81, 82, 85, 86].

Peer support interventions appeared to be acceptable and valued by some older people. They were seen as an acceptable way of gaining support and sharing information or advice. Participants mostly viewed peer support as feasible as it could be delivered through several different modalities (for example, face to face, in groups or online) that would fit individual preferences and lifestyles. However, it was clear that peer support was difficult to find and access in some settings, although appeared to be valued as a component of overall self-management of a CPLBP experience (low confidence) [77, 78, 80, 92, 96] [77, 78, 80, 92, 96].. The summary of the findings is presented in Table 6.

**Table 6:** Summary of qualitative findings table: Acceptability, values, and preferences for format of interventions and educational materials for CPLBP in older people

#	Summarised review finding	GRADE-CERQual Assessment of confidence	Explanation of GRADE-CERQual Assessment	References
10	Many participants liked a group format for physical exercise classes as these facilitated social support, collaborative learning and social activities which encouraged increased attendance. Participants in one study had a preference for shorter sessions on specific days to fit with their daily schedule.	Moderate confidence	Minor concerns regarding methodological limitations, Minor concerns regarding coherence, Minor concerns regarding adequacy, and Minor concerns regarding relevance	Hay M E & Connelly D M 2020; Dima A et al. 2013; Kuss K et al. 2016; Igwesi-Chidobe C N et al. 2020; Igwesi-Chidobe C N et al. 2019; Lee T L et al. 2020;
11	Participants broadly had positive views of peer support although they found it was difficult to access and did not know of support groups in their area. Empathy and “being believed” through common experience were the most important attributes in a peer supporter. Participants believed it would be helpful to share information and receive or exchange support and advice.	Low confidence	Moderate concerns regarding methodological limitations, Minor concerns regarding coherence, Minor concerns regarding adequacy, and Moderate concerns regarding relevance	Cummings E C et al. 2017; Cooper K et al. 2017; Hay M E & Connelly D M 2020; MacKichan F et al. 2013; Teh Carrie F et al. 2009;
12	Participants wanted educational materials for physical interventions which had drawings and descriptions of the exercises. This made them more comprehensible, easier to follow and helpful for present and future reference.	Low confidence	Minor concerns regarding methodological limitations, No/Very minor concerns regarding coherence, Serious concerns regarding adequacy, and Serious concerns regarding relevance	Dima A et al. 2013; Kuss K et al. 2016; Igwesi-Chidobe C N et al. 2020; Igwesi-Chidobe C N et al. 2019; Leonhardt Corinna et al. 2017;

### Cost/resources related to CPLBP care in older people

Seven studies from five countries contributed to these findings (USA, Australia, England, Nigeria, and Sweden). Participants in three studies were all over 60 [53, 84, 91], two studies had participants with a mean or average age of 60 or older [75, 79] and two studies were from LMICs or vulnerable populations of which both were included based on a lowered age threshold [83, 88, 89]. In five of the studies most of the participants where women (55–100%) [53, 75, 79, 84, 91]. In one study there was an equal distribution between men and women [83]. In one study most participants were men (66%) [88, 89].

We found that cost and resources could be a barrier to accessing care for CPLBP for some participants. High costs (financial, time and travel) could render treatments inaccessible to participants or acts as a deterrent (moderate confidence) [53, 79, 83, 91]. Many also preferred health care providers near where they lived to minimise travel burden. However, some participants were willing to travel if a trusted or favoured provider relocated, or they wanted to explore new treatment options. Others chose to find a new practitioner closer to them in this situation (moderate confidence) [53, 75, 79, 83, 84, 88, 91]. The summary of the findings is presented in Table 7.

**Table 7:** Summary of qualitative findings table: Cost/resources related to CPLBP care in older people

#	Summarised review finding	GRADE-CERQual Assessment of confidence	Explanation of GRADE-CERQual Assessment	References
13	Some participants viewed burden related to the intervention (financial, time and travel) as a barrier to accessing care. High cost rendered treatment inaccessible or deterred them from trying to adjust or continue with a recommended treatment. For others, who had the financial means or were accessing publicly funded health care, cost was not discussed.	Moderate confidence	No/Very minor concerns regarding methodological limitations, No/Very minor concerns regarding coherence, Minor concerns regarding adequacy, and Minor concerns regarding relevance	Lyons Kevin J et al. 2013; Stensland M 2021; Dima A et al. 2013; Igwesi-Chidobe C N et al. 2017;
14	Many participants had a preference for health care providers who were in close proximity to where they lived. For some, this was due to their CPLBP limiting their ability to travel more than short distances due to pain. If services were located a distance away, they were perceived as insufficient, inaccessible or that the distance was a barrier to care. However, some participants were willing to travel if a trusted or favoured health care provider relocated, or they were exploring new treatment options. Others preferred to find a new practitioner close to where they lived.	Moderate confidence	No/Very minor concerns regarding methodological limitations, Minor concerns regarding coherence, Minor concerns regarding adequacy, and Minor concerns regarding relevance	Lyons Kevin J et al. 2013; Stensland M 2021; Allvin R et al. 2019; Dima A et al. 2013; Lin I et al. 2014; Igwesi-Chidobe C N et al. 2017; Kirby E R et al. 2014;

## Feasibility

Twelve studies from eight countries contributed to these findings (USA, Canada, UK, Australia, England, Scotland, Nigeria, Chile). Participants in seven studies were all over 60 [53, 77, 80, 84, 91, 95, 96]. Three studies had participants with a mean or average age of 60 or older [78, 79, 92] and two studies were from LMICs or vulnerable populations of which both were included based on a lowered age threshold [81–83]. In 10 of the studies most of the participants where women (62–100%) [53, 77–82, 84, 91, 95, 96]. In two studies there was about an equal distribution between men and women [83, 92].

Some participants found information about treatments difficult to access and wanted help finding it or navigating the information from a health or care worker or through a peer support system. They felt that this could help them make decisions (low confidence) [78, 79, 84, 92, 96].

Physical activity and/or exercise was used a part of a self-management strategy for many participants. Activities such as swimming and walking were often mentioned as being done in their own time and when it fit their schedule. Some participants adopted physical exercise, assistive products, or alternative forms of treatment to supplement the conventional treatments they were receiving or when they felt “conventional treatments” failed. However, some did not inform their health care providers about their self-management strategies or changes they had made (moderate confidence). The summary of findings is presented in Table 8.

**Table 8:** Summary of qualitative findings table: Feasibility

#	Summarised review finding	GRADE-CERQual Assessment of confidence	Explanation of GRADE-CERQual Assessment	References
15	Some participants found information about treatments difficult to access and assess on their own. They wanted help navigating the information they had found from a health or care provider or a peer support system in order to make a decision about treatment.	Low confidence	Minor concerns regarding methodological limitations, No/Very minor concerns regarding coherence, Moderate concerns regarding adequacy, and Moderate concerns regarding relevance	Cummings E C et al. 2017; Dima A et al. 2013; MacKichan F et al. 2013; Teh Carrie F et al. 2009; Kirby E R et al. 2014;
16	Some participants adopted physical exercise, physical supports, or alternative forms of treatment (e.g., traditional or herbal medicine) as part of their self-management approach to supplement “conventional treatments” or when “conventional treatments” failed or were insufficient. This was often viewed as ‘experimenting’ to find a solution. Some participants did not inform their health care provider about these changes.	Moderate confidence	Minor concerns regarding methodological limitations, No/Very minor concerns regarding coherence, No/Very minor concerns regarding adequacy, and Minor concerns regarding relevance	Lyons Kevin J et al. 2013; Stensland M 2021; Cooper K et al. 2017; Hay M E & Connelly D M 2020; Igwesi-Chidobe C N et al. 2017; MacKichan F et al. 2013; Rodriguez I et al. 2019; Igwesi-Chidobe C N et al. 2019; Teh Carrie F et al. 2009;

## Equity and human rights

Seven studies from six countries contributed to this finding (USA, Canada, UK, England, Scotland, and Sweden, Brazil). Participants in four studies were all over 60 [77, 80, 91, 93] and three studies had participants with a mean or average age of 60 or older [75, 79, 92]. In six of the studies most of the participants were women [75, 77, 79, 80, 91, 93]. In one study there was an equal distribution of men and women [92].

Some participants perceived age-related stigma or bias when accessing healthcare for their CPLBP. They reported feeling that they were treated differently, dismissed, or discriminated against because of their age. They felt they were not taken seriously. This perceived stigma could deter them from seeking further treatment. However, in other cases participants believed that they were taken more seriously as they aged (Low confidence). The summary of the finding is presented in Table 9.

**Table 9:** Summary of qualitative findings table: Equity

#	Summarised review finding	GRADE-CERQual Assessment of confidence	Explanation of GRADE-CERQual Assessment	References
17	Some participants felt that health care providers dismissed or minimized their CPLBP due to their age and often with ageist statements. They often felt that they were not taken seriously or “fobbed off”, being told that pain was a natural consequence of ageing, and they should just “live with it”. This could make them feel horrible or in some cases deter them from seeking further treatment. However, a few participants described being taken more seriously as they got older especially if they had an accompanying serious illness.	Low confidence	Moderate concerns regarding methodological limitations, No/Very minor concerns regarding coherence, Minor concerns regarding adequacy, and Moderate concerns regarding relevance	Lyons Kevin J et al. 2013; Makris Una E et al. 2015; Cooper K et al. 2017; Hay M E & Connelly D M 2020; Allvin R et al. 2019; Dima A et al. 2013; MacKichan F et al. 2013;

## Additions to the framework

To incorporate all the data we analysed we expanded the framework to include a section we labelled person centred care.

## Discussion

### Main findings

Based on this synthesis of qualitative evidence derived from more than 650 older participants across 22 studies with representation across a range of geographies and economic contexts, we identified that older people living with CPLBP express values and preferences for their care that relate to therapeutic encounters and the importance of therapeutic alliance, irrespective of the type of treatment offered or delivered, choice of intervention, and intervention delivery modalities. Older people with CPLBP value therapeutic encounters that validate, legitimise, and respect their pain experience; that consider their context holistically and prioritise their needs and preferences; that adopt a person-focused and tailored approach to care; and that are supported by interprofessional communication. Older people value care that provides benefit to them, that includes a suite of interventions beyond analgesic medicines alone, and that is financially and geographically accessible. These findings provide critical context to service delivery models for older people; formulation of recommendations for guidelines that relate to older people; and service considerations for the implementation of clinical guidelines into practice, particularly related to how health care workers interact with older people, with attention to potential age-related bias, and how components of care are delivered.

### Person-centred care for older adults living with CPLBP

Many older people felt that healthcare providers did not legitimise their pain and that pain was deprioritised relative to other health conditions. Musculoskeletal pain, including CPLBP, is a common experience in older people [98, 99] and a very frequent co-morbidity with other noncommunicable diseases [100]. Therefore, pain assessment is a key component of the WHO Integrated Care for Older People (ICOPE) assessment and care pathway [101]. Comorbidities more strongly associated with mortality or acute health declines can make it difficult for health professionals to prioritise symptoms of CPLBP in time-limited clinical encounters. There seems to be a difference between patient and care provider priorities when it comes to pain management and our findings point to the need to legitimise and respond to pain as this clearly is a priority for older people, consistent with recently reported evidence [55]. Our findings point to the importance of the therapeutic relationship and communication between older people and care providers to understand the impact of, and preference for, CPLBP care. Older people also experienced issues linked to equity during the therapeutic encounter. These could be expressed through ageism and stigma associated with CPLBP. Being told to ‘just live with it’, or the idea that CPLBP was an inevitable part of ageing were common and suggest a potential age-related bias among healthcare providers. Being aware of potential clinician bias related to chronic pain in older people is important, since ageism is associated with poorer health outcomes, particularly in low resource settings [102].

The needs and priorities of older people may well differ to younger adults (e.g. return to work, taking care of dependents, intensity of everyday activities or sport may be less important for older people). There are previous findings of the perceived needs of adult groups with CPLBP [103, 104]. Consistent with other reviews among adults, we identified that older people value clear and consistent information, a clear diagnosis, prognosis, and a communication style that is meaningful and avoids jargon [105]. Communication that emphasises disability or impairments can be unhelpful to fostering pain self-efficacy, contribute to fear, unhelpful care seeking and further compound disability [106–109], which will foster healthy ageing. Rather, providing empowering and positive communication that is validating, helping to make sense of pain and the likelihood of a positive prognosis, providing cognitive reassurance and clear information about benefits and harms of interventions (in particular, medicines) can support shared decision-making, positive behaviour change towards effective self-management, and better engagement in meaningful activities [110]; all important for supporting healthy ageing.

We identified a preference for integrated and coordinated CPLBP care across care providers and facilities, consistent with the WHO ICOPE model [101]. This includes holistic care planning with comprehensive assessments and care plans aligned with the person’s values, priorities and preferences concerning their care. The older person should be involved with decision-making and goal-setting from the the start of their care journey. The care should be regular and include sustained follow-up, with integration and communication across different levels of care. This approach to care can help to avoid unnecessary treatments, polypharmacy and other potential harms [47, 110]. Our findings about fears of side effects, dependency and medicine withdrawal or non-adherence also points to the need for clinicians to take time to explain risk-benefits of different medicines so that older people understand what medicines are for and how to use them safely.

Values and preferences were largely agnostic to intervention modality, other than values relating to medicines, where specific issues related to fear of adverse events were observed. Although analgesic medicines were considered important for CPLBP care, older people preferred care packages that extend beyond analgesia so that care is more holistic and considers safety (e.g. issues of dependency for opioid analgesics) and that were meaningful and personally enjoyable – such as social benefits of group exercise. Recent evidence points to the importance of considering pharmacologic and non-pharmacologic therapies for CPLBP care, consistent with the experiences, values and preferences of older people [97]. Other evidence highlights care needs also extend beyond biomedical domains [24, 103]. Specifcally, tailoring components of care that addresses pain, emotional and social wellbeing, consistent with WHO ICOPE [101] model for improving functional ability, is important.

### Implementing and delivering care for older people living with CPLBP

When developing, implementing, and delivering interventions for older people who experience functional disability related to musculoskeletal pain (or other co-morbidities), consideration of economic, social, and cultural contexts is critical. Many experienced financial and geographic barriers to care. Access to care that is expensive (or not included in UHC or insurance rebates), that requires travel, or accessing buildings that are not adapted for people experiencing functional disability can be problematic. This threat is more severe for those living in poverty without access to healthcare or who cannot afford to access healthcare near them, such as in low-resource settings. This lack of access may lead to worse outcomes for older people living in these settings, widening inequities in access to health care and health outcomes. Services also need to consider the user’s social context [111]. If not taken into account, pain care is likely to be inequitable and inaccessible. Support needs to go beyond the purely biomedical (especially focusing on medication) and encompass interventions that address peer support and socialization as well as issues around acceptability and stigma. Interventions should be tailored to local contexts to increase social and cultural approval. Some of the interventions included in this synthesis, such as exercise, were stigmatized in some settings [81–83]. Other research has also found that stigma can be associated with gender [112] or with interventions targeted at older people [113].

Older people also wanted support for the implementation of interventions such as guidance on how to perform exercises in the form of drawings and text. None of the studies we included talked about digital supports except for those related to peer support where digital meetings were discussed. While some formative evidence exists around the role of digital technologies to support healthy ageing [114–116], further research is required to understand users’ perspectives, benefits and harms in different contexts and among different population groups. Other research has also shown the acceptability of peer support in older adults with CPLBP [117]. Research on older people has found that they access digital tools but may face barriers such as physical mobility, sight and hearing impairment and low digital literacy when trying to use them [118–120]. Studies examining the use of digital tools for interventions for low back pain not limited to older people have found that users value models that are easily understandable, provide an opening to further communication with health care providers, family and colleagues and can provide prompts, reassurance, ongoing support and interaction with other users [121, 122].

These empirical findings hold direct relevance to the formulation of recommendations in guidelines and implementation of recommended care within service models and local care pathways. In this context, the current QES has informed the development of the *WHO Guideline for non-surgical management of chronic primary low back pain in adults in primary and community care settings *[25]. Without consideration of the fundamental EtD factors (Table 2) and the evidence underpinning each when formulating recommendations for guidelines or implementation plans for service models, as presented in our QES, care recipients (and in some cases, care providers) may not accept or be able to access care, manifesting as an enduring disease burden and inequity in health outcomes. The QES findings, when coupled with evidence for benefit, harm, cost effectiveness and implementation feasibility and lived experience perspectives that contribute to co-creation of solutions (care recommendations, service models, care pathways) that are more likely to be implemented, sustainable and acceptable [123]. Indeed, consideration of qualitative evidence anchored to EtD domains is common practice for WHO guidelines [45].

### Implications for research

There was a clear lack of research from low- and middle-income settings as well as vulnerable populations in all settings. Most of the included studies explored the perceptions of community dwelling older adults. More research on the experiences of older adults living in residential care or other settings could help to broaden our understanding. Very few of the studies explored perceptions of specific interventions. Most looked at treatment across interventions and participants did not differentiate between interventions in the same way a health care provider would. For example, participants viewed the visit to the physiotherapist as the intervention whereas health care providers would view each of the treatments received as individual interventions. One topic not frequently discussed in the included studies was cost and out of pocket expenses. This may be because several studies were conducted as part of a trial where participants did not pay to access the intervention. Cost was also rarely discussed in studies taking place in publicly funded health care systems. Understanding affordability of care, willingness to pay and inequities in access to care due to cost will be important in planning implementation of health services for CPLBP care for older people. Further research is also needed on the perspectives and experiences of caregivers as there were no studies identified that explored this topic of interest.

### Implications for practice

The questions that form our implications for practice are derived from our findings with moderate or high confidence. They may help health system or program managers to plan, implement or manage interventions for CPLBP. It is important to consider local contextual factors including gender, age, cultural group, and education when implementing interventions.

- Is the burden to access services low (financial, time and travel)? Have issues related to burden and equity of access been considered?
- When planning, implementing, or managing an intervention for CPLBP or communicating with people over 60 with CPLBP:
  - ◦ have participants values and preferences been explored and taken into consideration?
  - ◦ are participants informed about the physical exercise or physical supports available to them?
- When communicating with adults over 60 with CPLBP, have values and preferences been considered, regarding:
  - ◦ communication, cultural preferences, and health care provider collaboration?
  - ◦ receiving a diagnosis and preferences for information?
- When prescribing medication, do health care workers provide open and honest communication with their patients about medications, the risk of side effects, and the risk of dependency, inviting them to return with concerns and informing of the importance of working together to manage their medications?

## Conclusion

Older people with CPLBP value therapeutic encounters that legitimise and respect their pain experience, that consider their context holistically and prioritises their needs and preferences, that is tailored, and that is supported by interprofessional communication. Older people value care that provides benefit, that includes interventions beyond analgesic medicines alone, and that is financially and geographically accessible. These findings provide critical context to the implementation of clinical guidelines and service models into practice, particularly related to how care providers interact with older people and how components of care are delivered and their accessibility.

### Supplementary Information

**Additional file 1. **Search strategy.

**Additional file 2. **Machine learning plan.

**Additional file 3. **Excluded full text studies with reason.

**Additional file 4. **Evidence profile table.

**Additional file 5. **Final adapted framework.

**Additional file 6. **ENTREQ Checklist.

## Data Availability

All the studies in this synthesis are published and available. The data that is in each finding is available in an interactive Summary of Qualitative Findings table. For access to this tool please send an email to the corresponding author.

## Abbreviations

CPLBP: Chronic primary low back pain
LBP: Low back pain
QES: Qualitative evidence synthesis
RCT: Randomised controlled trial
UHC: Universal health coverage
ICOPE: Integrated care for older people
WHO: World health organization
