# Supplementary material for: Acceptability, values, and preferences of older people for chronic low back pain management; a qualitative evidence synthesis
Source: BMC Geriatr. 2024 Jan 5;24:24. doi: 10.1186/s12877-023-04608-4 (PMC10768085; doi:10.1186/s12877-023-04608-4)
Supplement: Supplementary file 1 — Additional file 1. Search strategy. [file 12877_2023_4608_MOESM1_ESM.pdf]

Additional file 1: Search strategy

**CINAHL (EBSCO)** (Advanced search)<br>**Search date: 2022-04-28**		
**#**	**Search terms**	**Hits**
S1	(MH "Back Pain+")	33432
S2	(MH "Back Injuries+")	10682
S3	(MH "Coccyx/IN") OR (MH "Erector Spinae Muscles/IN") OR (MH "Intervertebral Disk/IN") OR (MH "Lumbar Vertebrae/IN") OR (MH "Lumbosacral Plexus/IN") OR (MH "Quadratus Lumborum Muscles/IN") OR (MH "Sacroiliac Joint/IN") OR (MH "Sacrum/IN") OR (MH "Spinal Canal+/IN") OR (MH "Spinal Nerve Roots/IN") OR (MH "Spine/IN") OR (MH "Vertebral Body/IN") OR (MH "Zygapophyseal Joint/IN")	2092
S4	(MH "Discitis")	54
S5	(MH "Intervertebral Disk Displacement")	4863
S6	(MH "Osteoarthritis, Spine")	90
S7	(MH "Piriformis Syndrome")	166
S8	(MH "Sciatica")	1821
S9	(MH "Spinal Diseases")	7412
S10	(MH "Spinal Osteophytosis+")	550
S11	(MH "Spinal Stenosis")	3049
S12	(MH "Spondylarthritis+")	7944
S13	(MH "Spondylitis, Ankylosing")	3662
S14	(MH "Spondylosis+")	2916
S15	TI ("ankylosing hyperostosis") OR AB ("ankylosing hyperostosis") OR SU ("ankylosing hyperostosis")	3
S16	TI ((((back OR spinal) N2 (ach* OR ailment* OR complaint* OR discomfort* OR disorder* OR injur* OR pain* OR sprain* OR strain*)) OR backpain OR backache)) OR AB ((((back OR spinal) N2 (ach* OR ailment* OR complaint* OR discomfort* OR disorder* OR injur* OR pain* OR sprain* OR strain*)) OR backpain OR backache)) OR SU ((((back OR spinal) N2 (ach* OR ailment* OR complaint* OR discomfort* OR disorder* OR injur* OR pain* OR sprain* OR strain*)) OR backpain OR backache))	74545
S17	TI ((((Bechterew* OR "Marie-Struempell") W0 disease) OR "spinal arthritis" OR spondylarthritis OR spondyloarthritis OR spondylitis)) OR AB ((((Bechterew* OR "Marie-Struempell") W0 disease) OR "spinal arthritis" OR spondylarthritis OR spondyloarthritis OR spondylitis)) OR SU ((((Bechterew* OR "Marie-Struempell") W0 disease) OR "spinal arthritis" OR spondylarthritis OR spondyloarthritis OR spondylitis))	6709
S18	TI (bertolotti* W0 syndrome) OR AB (bertolotti* W0 syndrome) OR SU (bertolotti* W0 syndrome)	30
S19	TI ((((coccy* OR tailbone OR "tail bone") N2 (ach* OR ailment* OR complaint* OR discomfort* OR disorder* OR injur* OR pain* OR sprain* OR strain*)) OR	168

	coccalg* OR coccydyn* OR coccygalg*)) OR AB ((((coccy* OR tailbone OR "tail bone") N2 (ach* OR ailment* OR complaint* OR discomfort* OR disorder* OR injur* OR pain* OR sprain* OR strain*)) OR coccalg* OR coccydyn* OR coccygalg*)) OR SU ((((coccy* OR tailbone OR "tail bone") N2 (ach* OR ailment* OR complaint* OR discomfort* OR disorder* OR injur* OR pain* OR sprain* OR strain*)) OR coccalg* OR coccydyn* OR coccygalg*))	
S20	TI (((disc? OR disk?) N3 (degenerat* OR degradat* OR displac* OR extru* OR hernia* OR injur* OR prolapse* OR protru* OR slipped))) OR AB (((disc? OR disk?) N3 (degenerat* OR degradat* OR displac* OR extru* OR hernia* OR injur* OR prolapse* OR protru* OR slipped))) OR SU (((disc? OR disk?) N3 (degenerat* OR degradat* OR displac* OR extru* OR hernia* OR injur* OR prolapse* OR protru* OR slipped)))	9739
S21	TI ((discitis OR diskitis OR spondyldiscitis OR spondyldiskitis OR spondylodiscitis OR spondylodiskitis)) OR AB ((discitis OR diskitis OR spondyldiscitis OR spondyldiskitis OR spondylodiscitis OR spondylodiskitis)) OR SU ((discitis OR diskitis OR spondyldiscitis OR spondyldiskitis OR spondylodiscitis OR spondylodiskitis))	797
S22	TI dorsalgia OR AB dorsalgia OR SU dorsalgia	36
S23	TI (((facet OR zygapophys*) N2 (ach* OR ailment* OR complaint* OR discomfort* OR disorder* OR injur* OR pain* OR sprain* OR strain*))) OR AB (((facet OR zygapophys*) N2 (ach* OR ailment* OR complaint* OR discomfort* OR disorder* OR injur* OR pain* OR sprain* OR strain*))) OR SU (((facet OR zygapophys*) N2 (ach* OR ailment* OR complaint* OR discomfort* OR disorder* OR injur* OR pain* OR sprain* OR strain*)))	529
S24	TI ((ischialgia OR lumboischialg* OR sciatica)) OR AB ((ischialgia OR lumboischialg* OR sciatica)) OR SU ((ischialgia OR lumboischialg* OR sciatica))	2386
S25	TI "kissing spine" OR AB "kissing spine" OR SU "kissing spine"	4
S26	TI (((lumb* OR thoracolumb*) N2 (ach* OR ailment* OR complaint* OR discomfort* OR disorder* OR pain* OR sprain* OR strain*))) OR AB (((lumb* OR thoracolumb*) N2 (ach* OR ailment* OR complaint* OR discomfort* OR disorder* OR pain* OR sprain* OR strain*))) OR SU (((lumb* OR thoracolumb*) N2 (ach* OR ailment* OR complaint* OR discomfort* OR disorder* OR pain* OR sprain* OR strain*)))	2810
S27	TI lumbago OR AB lumbago OR SU lumbago	148
S28	TI (((lumbar OR lumbosacral OR "lumbo-sacral" OR spinal) N5 stenosis)) OR AB (((lumbar OR lumbosacral OR "lumbo-sacral" OR spinal) N5 stenosis)) OR SU (((lumbar OR lumbosacral OR "lumbo-sacral" OR spinal) N5 stenosis))	4297
S29	TI (((osteoarthr* OR arthrosis) N3 (facet OR zygapophyseal OR "sacro-iliac" OR sacroiliac OR spinal OR spine))) OR AB (((osteoarthr* OR arthrosis) N3 (facet OR zygapophyseal OR "sacro-iliac" OR sacroiliac OR spinal OR spine))) OR SU (((osteoarthr* OR arthrosis) N3 (facet OR zygapophyseal OR "sacro-iliac" OR sacroiliac OR spinal OR spine)))	383

S30	TI "piriformis syndrome" OR AB "piriformis syndrome" OR SU "piriformis syndrome"	274
S31	TI ((("sacro-iliac joint" OR "sacroiliac joint" OR "SI joint") N2 (ach* OR ailment* OR complaint* OR discomfort* OR disorder* OR injur* OR pain* OR sprain* OR strain*))) OR AB ((("sacro-iliac joint" OR "sacroiliac joint" OR "SI joint") N2 (ach* OR ailment* OR complaint* OR discomfort* OR disorder* OR injur* OR pain* OR sprain* OR strain*))) OR SU ((("sacro-iliac joint" OR "sacroiliac joint" OR "SI joint") N2 (ach* OR ailment* OR complaint* OR discomfort* OR disorder* OR injur* OR pain* OR sprain* OR strain*)))	469
S32	TI sacroiliitis OR AB sacroiliitis OR SU sacroiliitis	552
S33	TI (sacrum N2 fracture*) OR AB (sacrum N2 fracture*) OR SU (sacrum N2 fracture*)	100
S34	TI (spinal N2 ankylos*) OR AB (spinal N2 ankylos*) OR SU (spinal N2 ankylos*)	126
S35	TI (((spinal OR spine) N3 deform*)) OR AB (((spinal OR spine) N3 deform*)) OR SU (((spinal OR spine) N3 deform*))	3637
S36	TI ((spinal N2 (ostechondrosis OR osteophytosis))) OR AB ((spinal N2 (ostechondrosis OR osteophytosis))) OR SU ((spinal N2 (ostechondrosis OR osteophytosis)))	328
S37	TI ((spondylarthropath* OR "spondyl-arthros*" OR spondylarthros* OR "spondylo-arthros*" OR spondyloarthros* OR spondylopath* OR spondylosis OR spondylolysis OR spondylolisthesis)) OR AB ((spondylarthropath* OR "spondyl-arthros*" OR spondylarthros* OR "spondylo-arthros*" OR spondyloarthros* OR spondylopath* OR spondylosis OR spondylolysis OR spondylolisthesis)) OR SU ((spondylarthropath* OR "spondyl-arthros*" OR spondylarthros* OR "spondylo-arthros*" OR spondyloarthros* OR spondylopath* OR spondylosis OR spondylolysis OR spondylolisthesis))	4622
S38	TI (((vertebra* OR spine OR spinal) N3 (collapse* OR fracture*))) OR AB (((vertebra* OR spine OR spinal) N3 (collapse* OR fracture*))) OR SU (((vertebra* OR spine OR spinal) N3 (collapse* OR fracture*)))	9760
S39	S1 OR S2 OR S3 OR S4 OR S5 OR S6 OR S7 OR S8 OR S9 OR S10 OR S11 OR S12 OR S13 OR S14 OR S15 OR S16 OR S17 OR S18 OR S19 OR S20 OR S21 OR S22 OR S23 OR S24 OR S25 OR S26 OR S27 OR S28 OR S29 OR S30 OR S31 OR S32 OR S33 OR S34 OR S35 OR S36 OR S37 OR S38	110742
S40	(MH "Chronic Disease+")	70065
S41	(MH "Chronic Pain")	25154

S42	TI (((chronic OR continual OR intractable OR lasting OR lingering OR "long-lasting" OR longlasting OR "long-standing" OR longstanding OR "long-term" OR longterm OR persist* OR prolonged OR recurrent OR refractory OR sustained) N5 (ach* OR backache OR backpain OR condition* OR disease* OR pain))) OR AB (((chronic OR continual OR intractable OR lasting OR lingering OR "long-lasting" OR longlasting OR "long-standing" OR longstanding OR "long-term" OR longterm OR persist* OR prolonged OR recurrent OR refractory OR sustained) N5 (ach* OR backache OR backpain OR condition* OR disease* OR pain))) OR SU (((chronic OR continual OR intractable OR lasting OR lingering OR "long-lasting" OR longlasting OR "long-standing" OR longstanding OR "long-term" OR longterm OR persist* OR prolonged OR recurrent OR refractory OR sustained) N5 (ach* OR backache OR backpain OR condition* OR disease* OR pain)))	244813
S43	S40 OR S41 OR S42	245637
S44	(MH "Interviews+")	240068
S45	(MH "Focus Groups")	48200
S46	(MH "Narratives+")	19731
S47	(MH "Qualitative Studies+")	168370
S48	TI ((("semi-structured" OR semistructured OR unstructured OR informal OR "in-depth" OR indepth OR "face-to-face" OR structured OR guide*) N2 (interview* OR discussion* OR questionnaire*))) OR AB ((("semi-structured" OR semistructured OR unstructured OR informal OR "in-depth" OR indepth OR "face-to-face" OR structured OR guide*) N2 (interview* OR discussion* OR questionnaire*))) OR SU ((("semi-structured" OR semistructured OR unstructured OR informal OR "in-depth" OR indepth OR "face-to-face" OR structured OR guide*) N2 (interview* OR discussion* OR questionnaire*)))	166545
S49	TI ((focus W0 group* OR qualitative OR ethnograph* OR fieldwork OR "field work" OR "key informant")) OR AB ((focus W0 group* OR qualitative OR ethnograph* OR fieldwork OR "field work" OR "key informant")) OR SU ((focus W0 group* OR qualitative OR ethnograph* OR fieldwork OR "field work" OR "key informant"))	240799
S50	TI "mixed method*" OR AB "mixed method*" OR SU "mixed method*"	22766
S51	S44 OR S45 OR S46 OR S47 OR S48 OR S49 OR S50	436658
S52	S39 AND S43 AND S51	1146

Ovid MEDLINE(R) ALL 1946 to April 27, 2022 (Advanced search)
Search date: 2022-04-28

#	Search terms	Hits
1	exp Back Pain/	42843
2	exp Back Injuries/	26666
3	exp Back Muscles/in [injuries]	55
4	Coccyx/in	103
5	Discitis/	2312
6	Intervertebral Disc Degeneration/	6872

7	Intervertebral Disc Displacement/	19669
8	exp Intervertebral Disc/in	532
9	Lumbar Vertebrae/in	5001
10	Lumbosacral Plexus/in	200
11	Lumbosacral Region/in	222
12	Osteoarthritis, Spine/	197
13	Piriformis Muscle Syndrome/	162
14	Sacroiliac Joint/in	393
15	Sacroiliitis/	650
16	Sacrum/in	1129
17	Sciatica/	5137
18	exp Spinal Canal/in	105
19	Spinal Diseases/	21983
20	Spinal Fractures/	16603
21	Spinal Injuries/	9010
22	exp Spinal Nerve Roots/in	1335
23	Spinal Osteochondrosis/	53
24	exp Spinal Osteophytosis/	4132
25	Spinal Stenosis/	6838
26	exp Spondylarthritis/	29105
27	Spondylitis/	4470
28	exp Spondylosis/	8289
29	Vertebral Body/in	10
30	Zygapophyseal Joint/in	165
31	ankylosing hyperostosis.ti,bt,ab,kf.	115
32	(((back or spinal) adj3 (ach* or ailment* or complaint* or discomfort* or disorder* or injur* or pain* or sprain* or strain*)) or backpain or backache).ti,bt,ab,kf.	121447
33	(((Bechterew* or Marie-Struempell) adj disease) or spinal arthritis or spondyl?arthritis or spondylitis).ti,bt,ab,kf.	23832
34	(bertolotti* adj syndrome).ti,bt,ab,kf.	66
35	(((coccy* or tailbone or tail bone) adj3 (ach* or ailment* or complaint* or discomfort* or disorder* or injur* or pain* or sprain* or strain*)) or coccalg* or coccydyn* or coccygalg*).ti,bt,ab,kf.	286
36	((disc? or disk?) adj4 (degenerat* or degradat* or displac* or extru* or hernia* or injur* or prolapse* or protru* or slipped)).ti,bt,ab,kf.	29138
37	(discitis or diskitis or spondyl?discitis or spondyl?diskitis).ti,bt,ab,kf.	3513
38	dorsalgia.ti,bt,ab,kf.	111
39	((facet or zygapophys*) adj3 (ach* or ailment* or complaint* or discomfort* or disorder* or injur* or pain* or sprain* or strain*)).ti,bt,ab,kf.	1029
40	(ischialgia or lumboischialg* or sciatica).ti,bt,ab,kf.	5275
41	kissing spine.ti,bt,ab,kf.	32
42	((lumb* or thoracolumb*) adj3 (ach* or ailment* or complaint* or discomfort* or disorder* or pain* or sprain* or strain*)).ti,bt,ab,kf.	7640
43	lumbago.ti,bt,ab,kf.	1444
44	((lumbar or lumbosacral or lumbo-sacral or spinal) adj6 stenosis).ti,bt,ab,kf.	8787
45	((osteoarthr* or arthrosis) adj4 (facet or zygapophyseal or sacro-iliac or sacroiliac or spinal or spine)).ti,bt,ab,kf.	894
46	piriformis syndrome.ti,bt,ab,kf.	329

47	((sacro-iliac or sacroiliac joint or SI joint) adj3 (ach* or ailment* or complaint* or discomfort* or disorder* or injur* or pain* or sprain* or strain*)).ti,bt,ab,kf.	703
48	sacroiliitis.ti,bt,ab,kf.	2268
49	(sacrum adj3 fracture*).ti,bt,ab,kf.	374
50	(spinal adj3 ankylos*).ti,bt,ab,kf.	394
51	((spinal or spine) adj4 deform*).ti,bt,ab,kf.	9245
52	(spinal adj3 (ostechondrosis or osteophytosis)).ti,bt,ab,kf.	58
53	(spondylarthropath* or spondyl-arthros* or spondylarthros* or spondylo-arthros* or spondyloarthros* or spondylopath* or spondylosis or spondylolysis or spondylolisthesis).ti,bt,ab,kf.	11620
54	((vertebra* or spine or spinal) adj4 (collapse* or fracture*)).ti,bt,ab,kf.	23406
55	or/1-54	271243
56	Chronic Disease/ or Chronic Pain/ or Intractable Pain/	300053
57	((chronic or continual or intractable or lasting or lingering or long-lasting or longlasting or long-standing or longstanding or long-term or longterm or persist* or prolonged or recurrent or refractory or sustained) adj6 (ach* or backache or backpain or condition* or disease* or pain)).ti,bt,ab,kf.	649647
58	Interviews as Topic/ or Focus Groups/ or Narration/ or Qualitative Research/ [University of Texas - Search filter for Qualitative Studies, MEDLINE]	153471
59	((("semi-structured" or semistructured or unstructured or informal or "in-depth" or indepth or "face-to-face" or structured or guide) adj3 (interview* or discussion* or questionnaire*)) or (focus group* or qualitative or ethnograph* or fieldwork or "field work" or "key informant") or mixed method*).ti,ab,kf. [University of Texas - Search filter for Qualitative Studies, MEDLINE – with minor revisions (added field code .kf and keyword "mixed method*") - https://libguides.sph.uth.tmc.edu/search_filters/ovid_medline_filters]	428164
60	55 and (56 or 57) and (58 or 59) [low back pain AND chronic pain AND qualitative research]	988
