# Supplementary material for: Acceptability, values, and preferences of older people for chronic low back pain management; a qualitative evidence synthesis
Source: BMC Geriatr. 2024 Jan 5;24:24. doi: 10.1186/s12877-023-04608-4 (PMC10768085; doi:10.1186/s12877-023-04608-4)
Supplement: Supplementary file 2 — Additional file 2. Machine learning plan. [file 12877_2023_4608_MOESM2_ESM.docx]

# Additional file 2: Machine learning plan

1. We ran the Cochrane RCT classifier to identify and automatically exclude the studies that were most likely to have a controlled design, without having to assess them. We excluded all those predicted to be RCTs with more than 80% likelihood. The Cochrane RCT classifier has been built, tested, and validated with tens of thousands of studies from the CENTRAL database and is a recommended practice for all Cochrane authors (1).
2. We ran the automatic text clustering feature in EPPI Reviewer (2) to identify clusters of studies that clearly did not meet our inclusion criteria (3). Automatic text clustering is an unsupervised machine learning approach to find patterns and similarities in the data and group like together, and unlike apart. These clusters reflect semantic meaning. Automatic text clustering in EPPI Reviewer is powered by Lingo3G. Possible clusters that did not meet our inclusion criteria included diagnostic categories, populations such as children or study designs indicated by clusters with labels such as non-randomized or survey. Clusters that indicated studies that did not meet our inclusion criteria were checked by HA or CHH and excluded.
3. We used priority screening in EPPI Reviewer. Priority screening is a ranking algorithm that continuously learns from researcher decisions of screening based on title and abstract text and pushes relevant studies to the front of the screening queue. It allows relevant studies to be identified and included almost immediately by researchers in the screening process; conversely, studies reserved for the end of the queue are very likely irrelevant (4, 5). Once we had no included studies for the last 100 items screened, we changed to single screening. After screening a further 150 items without including a study we stopped screening.

1. Thomas J, McDonald S, Noel-Storr A, Shemilt I, Elliott J, Mavergames C, et al. Machine learning reduced workload with minimal risk of missing studies: development and evaluation of a randomized controlled trial classifier for cochrane reviews. Journal of clinical epidemiology. 2021;133:140-51.

2. Thomas J, Brunton J, Graziosi S. EPPI-Reviewer 4: software for research synthesis. EPPI-Centre Software. London: Social Science Research Unit, UCL Institute of Education. 2010. 2018.

3. Muller AE, Ames HMR, Jacobsen Jardim PS, Rose CJ. Machine learning in systematic reviews: comparing automated text clustering with Lingo3G and human researcher categorization in a rapid review. Research Synthesis Methods. 2021.

4. Gates A, Guitard S, Pillay J, Elliott SA, Dyson MP, Newton AS, et al. Performance and usability of machine learning for screening in systematic reviews: a comparative evaluation of three tools. Systematic reviews. 2019;8(1):1-11.

5. Muller AE, Ames HMR, Himmels JPW, Jardim PSJ, Nguyen HL, Rose CJ, et al. Implementation of machine learning in evidence syntheses in the Cluster for Reviews and Health Technology Assessments: Final report 2020-2021. 2021.
