# Supplementary material for: Acceptability, values, and preferences of older people for chronic low back pain management; a qualitative evidence synthesis
Source: BMC Geriatr. 2024 Jan 5;24:24. doi: 10.1186/s12877-023-04608-4 (PMC10768085; doi:10.1186/s12877-023-04608-4)
Supplement: Supplementary file 3 — Additional file 3. Excluded full text studies with reason. [file 12877_2023_4608_MOESM3_ESM.docx]

# Additional file 3: Excluded full text studies with reason

## Wrong target group (n=117)

Alhowimel A, Alotaibi M and Coulson N ; Radford K ; (2020) Psychosocial consequences of diagnosing nonspecific low-back pain radiologically: a qualitative study. Physiotherapy Theory & Practice , 1-7

Allegretti A, Borkan J and Reis S ; Griffiths F ; (2010) Paired interviews of shared experiences around chronic low back pain: classic mismatch between patients and their doctors. Family Practice 27(6), 676-83

Alnojeidi A H and Capo-Lugo C E; Sturgeon J A; Trost Z (2021) Injustice Through Cultural Lens: A Pilot Qualitative Exploration of Pain-Related Injustice Appraisals Among Arab-Americans with Chronic Low Back Pain. Journal of Pain 27, 27

Alves Martins and José (2017) Socioanthropological contributions to physical therapy in chronic back pain. Fisioterapia em Movimento 30(Sepp1), S101-S109

Angel S, Jensen L D and Maribo T ; Gonge B K; Buus N ; (2017) Narratives of life with long-term low back pain: A follow up interview study. Scandinavian Journal of Pain 17, 382-389

Ashby Samantha, Fitzgerald Maureen and Raine Simon ; (2012) The impact of chronic low back pain on leisure participation: implications for occupational therapy. British Journal of Occupational Therapy 75(11), 503-508

Banbury P, Feenan K and Allcock N ; (2008) Experiences of analgesic use in patients with low back pain. British Journal of Nursing 17(19), 1215-8

Bath Brenna, Jaindl Bryna and Dykes Lorenne ; Coulthard Jason ; Naylen Jessica ; Rocheleau Noelle ; Clay Lynne ; Khan Muhammad I; Trask Catherine ; (2019) Get 'Er Done: Experiences of Canadian Farmers Living with Chronic Low Back Disorders. Physiotherapy Canada 71(1), 24-33

Bogdal J, Schmidt A M and Nielsen K O; Handberg C ; (2021) An Integrated Multidisciplinary Rehabilitation Program Experienced by Patients with Chronic Low Back Pain. Clinical Medicine & Research 19(4), 192-202

Bourke M J, Ferguson D and Cooke M ; (2022) Patient Experiences of Self-Management for Chronic Low Back Pain: A Qualitative Study. Physical Therapy 30, 30

Boutevillain L, Dupeyron A and Rouch C ; Richard E ; Coudeyre E ; (2017) Facilitators and barriers to physical activity in people with chronic low back pain: A qualitative study. PLoS ONE [Electronic Resource] 12(7), e0179826

Braeuninger-Weimer K, Anjarwalla N and Pincus T ; (2019) Discharged and dismissed: A qualitative study with back pain patients discharged without treatment from orthopaedic consultations. European Journal of Pain 23(8), 1464-1474

Briggs A M, Slater H and Bunzli S ; Jordan J E; Davies S J; Smith A J; Quintner J L; (2012) Consumers' experiences of back pain in rural Western Australia: access to information and services, and self-management behaviours. BMC Health Services Research 12, 357

Buchman D Z, Ho A and Illes J ; (2016) You Present like a Drug Addict: Patient and Clinician Perspectives on Trust and Trustworthiness in Chronic Pain Management. Pain Medicine 17(8), 1394-406

Bunzli S, Smith A and Schutze R ; O'Sullivan P ; (2015) Beliefs underlying pain-related fear and how they evolve: a qualitative investigation in people with chronic back pain and high pain-related fear. BMJ Open 5(10), e008847

Bunzli S, McEvoy S and Dankaerts W ; O'Sullivan P ; O'Sullivan K ; (2016) Patient Perspectives on Participation in Cognitive Functional Therapy for Chronic Low Back Pain. Physical Therapy 96(9), 1397-407

Caeiro C, Moore A and Price L ; (2016) Portuguese individuals' experiences and perspectives of non-specific chronic low back pain: An interpretative phenomenological analysis. Manual Therapy 25, e149-e149

Caeiro C, Moore A and Price L ; (2021) Clinical encounters may not be responding to patients' search for meaning and control over non-specific chronic low back pain - an interpretative phenomenological analysis. Disability & Rehabilitation , 1-15

Caiata Zufferey, M and Schulz P J; (2009) Self-management of chronic low back pain: an exploration of the impact of a patient-centered website. Patient Education & Counseling 77(1), 27-32

Campbell C and Guy A (2007) 'Why can't they do anything for a simple back problem?' A qualitative examination of expectations for low back pain treatment and outcome. Journal of Health Psychology 12(4), 641-652

Cedraschi C, Robert J and Perrin E ; Fischer W ; Goerg D ; Vischer T L; (1996) The role of congruence between patient and therapist in chronic low back pain patients. Journal of Manipulative & Physiological Therapeutics 19(4), 244-9

Cedraschi C, Luthy C and Girard E ; Piguet V ; Desmeules J ; Allaz A F; (2012) Representations of symptom history in women with fibromyalgia vs chronic low back pain: a qualitative study. Pain Medicine 13(12), 1562-70

Chew C A and May C R; (1997) The benefits of back pain. Family Practice 14(6), 461-5

Combs M A and Thorn B E; (2014) Barriers and facilitators to yoga use in a population of individuals with self-reported chronic low back pain: a qualitative approach. Complementary Therapies in Clinical Practice 20(4), 268-75

Cook F M and Hassenkamp A (2000) Active rehabilitation for chronic low back pain: the patient's perspective. Physiotherapy 86(2), 61-68

Cooper K, Smith B H and Hancock E ; (2008) Patient-centredness in physiotherapy from the perspective of the chronic low back pain patient. Physiotherapy 94(3), 244-252

Cooper K, Smith B H and Hancock E ; (2009) Patients' perceptions of self-management of chronic low back pain: evidence for enhancing patient education and support. Physiotherapy 95(1), 43-50

Crowe M, Whitehead L and Gagan M J; Baxter G D; Pankhurst A ; Valledor V ; (2010) Listening to the body and talking to myself - the impact of chronic lower back pain: a qualitative study. International Journal of Nursing Studies 47(5), 586-92

Crowe M, Whitehead L and Jo Gagan ; M ; Baxter D ; Panckhurst A ; (2010) Self-management and chronic low back pain: a qualitative study. Journal of Advanced Nursing 66(7), 1478-86

Darlow B, Dowell A and Baxter G D; Mathieson F ; Perry M ; Dean S ; (2013) The enduring impact of what clinicians say to people with low back pain. Annals of Family Medicine 11(6), 527-34

De Sola H, Maquibar A and Failde I ; Salazar A ; Goicolea I ; (2020) Living with opioids: A qualitative study with patients with chronic low back pain. Health Expectations 23(5), 1118-1128

Doran Natasha J (2014) Experiencing Wellness Within Illness: Exploring a Mindfulness-Based Approach to Chronic Back Pain. Qualitative Health Research 24(6), 749-760

Dunleavy K, Bishop M and Coffman A ; Reidy J ; Kane A ; (2021) Chronic lower back pain in aquaculture clam farmers: adoption and feasibility of self-management strategies introduced using a rapid prototype participatory ergonomic approach. International Journal of Occupational Safety & Ergonomics , 1-11

Eaves E R, Sherman K J; Ritenbaugh C and Hsu C ; Nichter M ; Turner J A; Cherkin D C; (2015) A qualitative study of changes in expectations over time among patients with chronic low back pain seeking four CAM therapies. BMC Complementary & Alternative Medicine 15, 12

Ellegaard H and Pedersen B D (2012) Stress is dominant in patients with depression and chronic low back pain. A qualitative study of psychotherapeutic interventions for patients with non-specific low back pain of 3-12 months' duration. BMC Musculoskeletal Disorders 13, 166

Escolar-Reina P, Medina-Mirapeix F and Gascon-Canovas J J; Montilla-Herrador J ; Jimeno-Serrano F J; de Oliveira Sousa S. L; del Bano-Aledo M E; Lomas-Vega R ; (2010) How do care-provider and home exercise program characteristics affect patient adherence in chronic neck and back pain: a qualitative study. BMC Health Services Research 10, 60

Evers S, Hsu C and Sherman K J; Balderson B ; Hawkes R ; Brewer G ; La Porte A M; Yeoman J ; Cherkin D ; (2017) Patient Perspectives on Communication with Primary Care Physicians about Chronic Low Back Pain. Permanente Journal 21, 16-177

Feldman R, Nudelman Y and Haleva-Amir S ; Ben Ami ; N ; (2021) Patients' prior perceptions and expectations of the Enhanced Transtheoretical Model Intervention for chronic low back pain: A qualitative study. Musculoskeletal Care 02, 02

Fu Y, McNichol E and Marczewski K ; Closs S J; (2016) Exploring the Influence of Patient-Professional Partnerships on the Self-Management of Chronic Back Pain: A Qualitative Study. Pain Management Nursing 17(5), 339-49

Fu Y, McNichol E and Marczewski K ; Jose Closs ; S ; (2018) The Management of Chronic Back Pain in Primary Care Settings: Exploring Perceived Facilitators and Barriers to the Development of Patient-Professional Partnerships. Qualitative Health Research 28(9), 1462-1473

Fu Y, Yu G and McNichol E ; Marczewski K ; Closs S J; (2018) The association between patient-professional partnerships and self-management of chronic back pain: A mixed methods study. European Journal of Pain 22(7), 1229-1244

Gaskell L and Williams A E (2019) A qualitative study of the experiences and perceptions of adults with chronic musculoskeletal conditions following a 12-week Pilates exercise programme. Musculoskeletal Care 17(1), 54-62

Gladwell P W, Badlan K and Cramp F ; Palmer S ; (2015) Direct and Indirect Benefits Reported by Users of Transcutaneous Electrical Nerve Stimulation for Chronic Musculoskeletal Pain: Qualitative Exploration Using Patient Interviews. Physical Therapy 95(11), 1518-28

Gladwell P W, Badlan K and Cramp F ; Palmer S ; (2016) Problems, Solutions, and Strategies Reported by Users of Transcutaneous Electrical Nerve Stimulation for Chronic Musculoskeletal Pain: Qualitative Exploration Using Patient Interviews. Physical Therapy 96(7), 1039-48

Glenton C (2002) Developing patient-centred information for back pain sufferers. Health Expectations 5(4), 319-329

Glenton C, Nilsen E S and Carlsen B ; (2006) Lay perceptions of evidence-based information--a qualitative evaluation of a website for back pain sufferers. BMC Health Services Research 6, 34

Gonzalez A I, Ramtin S and Ring D ; Donthula D ; Queralt M ; (2022) People Have Mixed Reactions to Both Physiological and Psychological Explanations of Disproportionate Pain. Clinical Orthopaedics & Related Research 08, 08

Harman K, Macrae M and Vallis M ; Bassett R ; (2014) Working with people to make changes: a behavioural change approach used in chronic low back pain rehabilitation. Physiotherapy Canada 66(1), 82-90

Hjertstrand J, Palmgren P J and Axen I ; Eklund A ; (2021) The nordic maintenance care program: patient experience of maintenance care-a qualitative study. Chiropractic & manual therapies 29(1), 28

Holloway I, Sofaer-Bennett B and Walker J ; (2007) The stigmatisation of people with chronic back pain. Disability & Rehabilitation 29(18), 1456-1464

Holopainen R, Piirainen A and Heinonen A ; Karppinen J ; O'Sullivan P ; (2018) From "Non-encounters" to autonomic agency. Conceptions of patients with low back pain about their encounters in the health care system. Musculoskeletal Care 16(2), 269-277

Honore Grauslund, A M and Solmunde Michelsen J; Esbensen B A; (2021) Everyday life with chronic back pain: a qualitative study among Turkish immigrants in Denmark. Disability & Rehabilitation 43(8), 1162-1170

Hopton A, Thomas K and MacPherson H ; (2013) The acceptability of acupuncture for low back pain: a qualitative study of patient's experiences nested within a randomised controlled trial. PLoS ONE [Electronic Resource] 8(2), e56806

Howarth M L (2012) Being believed and believing in: the impact of delegitimation on person centred care for people with chronic back pain. , University of Salford (United Kingdom).

Howarth M, Warne T and Haigh C ; (2014) Pain from the inside: understanding the theoretical underpinning of person-centered care delivered by pain teams. Pain Management Nursing 15(1), 340-8

Hsu C, Sherman K J and Eaves E R; Turner J A; Cherkin D C; Cromp D ; Schafer L ; Ritenbaugh C ; (2014) New perspectives on patient expectations of treatment outcomes: results from qualitative interviews with patients seeking complementary and alternative medicine treatments for chronic low back pain. BMC Complementary & Alternative Medicine 14, 276

Haanstra T M, Hanson L and Evans R ; van Nes F A; De Vet H C; Cuijpers P ; Ostelo R W; (2013) How do low back pain patients conceptualize their expectations regarding treatment? Content analysis of interviews. European Spine Journal 22(9), 1986-95

Igwesi-Chidobe C N, Kitchen S and Sorinola I O; Godfrey E L; (2017) "A life of living death": the experiences of people living with chronic low back pain in rural Nigeria. Disability & Rehabilitation 39(8), 779-790

Igwesi-Chidobe C N, Godfrey E L; Kitchen S and Onwasigwe C N; Sorinola I O; (2019) Community-based self-management of chronic low back pain in a rural African primary care setting: a feasibility study. Primary Health Care Research & Development 20, e45

Igwesi-Chidobe C N, Kitchen S and Sorinola I O; Godfrey E L; (2020) Evidence, theory and context: using intervention mapping in the development of a community-based self-management program for chronic low back pain in a rural African primary care setting - the good back program. BMC Public Health 20(1), 343

Jyung H, Mah D M and Moonaz S ; Rai M ; Bhandiwad A ; Nielsen A ; Teets R ; (2022) "The Pain Left, I Was Off and Running": A Qualitative Analysis of Group Acupuncture and Yoga Therapy for Chronic Pain in a Low-Income and Ethnically Diverse Population. Journal of Integrative and Complementary Medicine 28(4), 328-338

Karran E L and Fryer C E; Middleton J W; Moseley G L; (2022) Exploring the social determinants of health outcomes for adults with low back pain or spinal cord injury and persistent pain: a mixed methods study. Journal of Pain 13, 13

Kawi J (2014) Chronic low back pain patients' perceptions on self-management, self-management support, and functional ability. Pain Management Nursing 15(1), 258-64

King R, Robinson V and Elliott-Button H L; Watson J A; Ryan C G; Martin D J; (2018) Pain Reconceptualisation after Pain Neurophysiology Education in Adults with Chronic Low Back Pain: A Qualitative Study. Pain Research & Management 2018, 3745651

Knish S and Calder P (1999) Beliefs of chronic low back pain sufferers: a concept map. Canadian Journal of Rehabilitation 12(3), 165-177

Korsch S, Herbold D and Wiezoreck M ; Geigner B ; Beddies A ; Worringen U ; Hampel P ; (2016) [Promoting Factors, Barriers and Barrier Management to the Implementation of Health-Promoting Behavior among Rehabilitative Patients with Chronic Low Back Pain - A Qualitative Analysis]. Rehabilitation 55(4), 210-6

Laerum E, Indahl A and Skouen J S; (2006) What is "the good back-consultation"? A combined qualitative and quantitative study of chronic low back pain patients' interaction with and perceptions of consultations with specialists. Journal of Rehabilitation Medicine 38(4), 255-62

Lansing J E, Ellingson L D; DeShaw K J; Cruz-Maldonado G and Hurt T R; Meyer J D; (2021) A qualitative analysis of barriers and facilitators to reducing sedentary time in adults with chronic low back pain. BMC Public Health 21(1), 215

Liddle S D and Baxter G D; Gracey J H; (2007) Chronic low back pain: patients' experiences, opinions and expectations for clinical management. Disability & Rehabilitation 29(24), 1899-1909

Lillrank A (2003) Back pain and the resolution of diagnostic uncertainty in illness narratives. Social Science & Medicine 57(6), 1045-54

Lin I B, O'Sullivan P B; Coffin J A; Mak D B; Toussaint S and Straker L M; (2013) Disabling chronic low back pain as an iatrogenic disorder: a qualitative study in Aboriginal Australians. BMJ Open 3(4),

Lin I, O'Sullivan P and Coffin J ; Mak D B; Toussaint S ; Straker L ; (2014) 'I can sit and talk to her': Aboriginal people, chronic low back pain and healthcare practitioner communication. Australian Family Physician 43(5), 320-4

Lin T A. Y. Hui and Hong-Gu H E; Lau Lydia Siew Tiang; Hui T A. Y. Kwang; Tsen Marie Li Mee; Chan Sally Wai Chi; (2014) Research in brief -- The experiences of older adults with chronic low back pain: A descriptive qualitative study. Singapore Nursing Journal 41(2), 45-47

Lindgreen P, Rolving N and Nielsen C V; Lomborg K ; (2016) Interdisciplinary Cognitive-Behavioral Therapy as Part of Lumbar Spinal Fusion Surgery Rehabilitation: Experience of Patients With Chronic Low Back Pain. Orthopaedic Nursing 35(4), 238-47

Lovo S, Harrison L and O'Connell M E; Trask C ; Bath B ; (2019) Experience of patients and practitioners with a team and technology approach to chronic back disorder management. Journal of multidisciplinary healthcare 12, 855-869

MacPherson H, Thorpe L and Thomas K ; (2006) Beyond needling -- therapeutic processes in acupuncture care: a qualitative study nested within a low-back pain trial. Journal of Alternative & Complementary Medicine 12(9), 873-880

May C R and Rose M J; Johnstone F C; (2000) Dealing with doubt. How patients account for non-specific chronic low back pain. Journal of Psychosomatic Research 49(4), 223-5

Mbarga J, Foley R A and Pichonnaz C ; Ancey C ; (2020) Trajectoires de personnes souffrant de lombalgie chronique : ruptures et reconstructions après un programme de rééducation. Sante Publique (Vandoeuvre-Les-Nancey). Vol 32(1), 19-28

McPhillips-Tangum C A and Cherkin D C; Rhodes L A; Markham C (1998) Reasons for repeated medical visits among patients with chronic back pain. Journal of General Internal Medicine 13(5), 289-95

Medina-Mirapeix F, Escolar-Reina P and Gascon-Canovas J J; Montilla-Herrador J ; Collins S M; (2009) Personal characteristics influencing patients' adherence to home exercise during chronic pain: a qualitative study. Journal of Rehabilitation Medicine 41(5), 347-52

Merolli M, Marshall C J and Pranata A ; Paay J ; Sterling L ; (2019) User-Centered Value Specifications for Technologies Supporting Chronic Low-Back Pain Management. Studies in Health Technology & Informatics 264, 1288-1292

Miller J and Timson D (2004) Exploring the experience of partners who live with a chronic low back pain sufferer. Health & Social Care in the Community 12(1), 34-42

Morris A L (2004) Patients' perspectives on self-management following a back rehabilitation programme. Musculoskeletal Care 2(3), 165-179

Myburgh C, Larsen T B and Kjaer P ; (2022) 'When the picture does not really tell the story'- A qualitative exploration of the MRI report of findings as a means for generating shared diagnostic meaning during the management of patients suffering from persistent spinal pain. Patient Education & Counseling 105(1), 221-227

Nagl M, Ullrich A and Farin E ; (2013) [Comprehensibility of patient education in orthopaedic rehabilitation: a qualitative study on patients and providers]. Rehabilitation 52(1), 34-9

Nichols V P, Griffiths F E; Patel S and Lamb S E; (2015) Patterns of interaction between factors that enhance or inhibit recovery from chronic low back pain. Disability & Rehabilitation 37(3), 194-206

Oien A M, Iversen S and Stensland P ; (2007) Narratives of embodied experiences - therapy processes in Norwegian psychomotor physiotherapy. Advances in Physiotherapy 9(1), 31-39

Orrock P J (2016) The patient experience of osteopathic healthcare. Manual Therapy 22, 131-7

Palazzo C, Klinger E and Dorner V ; Kadri A ; Thierry O ; Boumenir Y ; Martin W ; Poiraudeau S ; Ville I ; (2016) Barriers to home-based exercise program adherence with chronic low back pain: Patient expectations regarding new technologies. Annals of Physical & Rehabilitation Medicine 59(2), 107-13

Petrozzi M J, Spencer G and Mackey M G; (2021) A process evaluation of the Mind Your Back trial examining psychologically informed physical treatments for chronic low back pain. Chiropractic & manual therapies 29(1), 32

Pires D, Cruz E B and Costa D ; Nunes C ; (2022) Beyond pain and disability: an explanatory mixed methods study exploring outcomes after physiotherapy intervention in patients with chronic low back pain. Disability & Rehabilitation 44(6), 882-891

Plank A, Rushton A and Ping Y ; Mei R ; Falla D ; Heneghan N R; (2021) Exploring expectations and perceptions of different manual therapy techniques in chronic low back pain: a qualitative study. BMC Musculoskeletal Disorders 22(1), 444

Pugh J D and Williams A M; (2014) Feldenkrais method empowers adults with chronic back pain. Holistic Nursing Practice 28(3), 171-83

Rajan P, Hiller C E and Leaver A ; Dennis S ; Refshauge K ; Brady B ; (2022) Pain experiences of Hindi-speaking Indian migrants in Sydney: a qualitative study. Physiotherapy 116, 25-32

Randhawa Sarkaw Mohammad and Hay-Smith E Jean C; Grainger Rebecca (2019) The experience of lower back pain and its treatment among paramedics in New Zealand: a qualitative study. Australasian Journal of Paramedicine 16, 1-7

Reid M (2004) An assessment of health needs of chronic low back pain patients from general practice. Journal of Health Psychology 9(3), 451-462

Rhodes L A, McPhillips-Tangum C A; Markham C and Klenk R ; (1999) The power of the visible: the meaning of diagnostic tests in chronic back pain. Social Science & Medicine 48(9), 1189-1203

Riipinen P, Holmes M and Ogilvie S ; Newell D ; Byfield D ; du Rose ; A ; (2022) Patient's perception of exercise for management of chronic low back pain: A qualitative study exercise for the management of low back pain. Musculoskeletal Care 06, 06

Rostami K, Sharif F and Zarshenas L ; Ebadi A ; Farbood A ; (2019) Health Needs in Patients Suffering from Chronic Back Pain: A Qualitative Study. Anesthesiology & Pain Medicine 9(2), e85244

Saner J, Bergman E M and de Bie R A; Sieben J M; (2018) Low back pain patients' perspectives on long-term adherence to home-based exercise programmes in physiotherapy. Musculoskeletal Science & Practice 38, 77-82

Skelton A M and Murphy E A; Murphy R J; O'Dowd T C; (1996) Patients' views of low back pain and its management in general practice. British Journal of General Practice 46(404), 153-6

Slade S C, Molloy E and Keating J L; (2009) Stigma experienced by people with nonspecific chronic low back pain: a qualitative study. Pain Medicine 10(1), 143-54

Slade S C, Molloy E and Keating J L; (2009) People with non-specific chronic low back pain who have participated in exercise programs have preferences about exercise: a qualitative study. Australian Journal of Physiotherapy 55(2), 115-121

Sloots M, Scheppers E F and Bartels E A; Dekker J H; Geertzen J H; Dekker J ; (2009) First rehabilitation consultation in patients of non-native origin: factors that lead to tension in the patient-physician interaction. Disability & Rehabilitation 31(22), 1853-61

Sloots M, Dekker J H and Pont M ; Bartels E A; Geertzen J H; Dekker J ; (2010) Reasons of drop-out from rehabilitation in patients of Turkish and Moroccan origin with chronic low back pain in The Netherlands: a qualitative study. Journal of Rehabilitation Medicine 42(6), 566-73

Snelgrove S, Edwards S and Liossi C ; (2013) A longitudinal study of patients' experiences of chronic low back pain using interpretative phenomenological analysis: changes and consistencies. Psychology & Health 28(2), 121-38

Sokunbi O, Watt P and Moore A ; (2008) Experiences of patients with chronic low back disorder of the use of spinal stabilisation exercises--a qualitative study. Nigerian Quarterly Journal of Hospital Medicine 18(4), 231-43

Sokunbi O, Cross V and Watt P ; Moore A ; (2010) Experiences of individuals with chronic low back pain during and after their participation in a spinal stabilisation exercise programme - a pilot qualitative study. Manual Therapy 15(2), 179-84

Stenner R (2014) Exercise prescription for patients with non-specific chronic low back pain: a qualitative exploration of physiotherapy practice. , University of the West of England, Bristol (United Kingdom).

Stenner R, Swinkels A and Mitchell T ; Palmer S ; (2016) Exercise prescription for non-specific chronic low back pain (NSCLBP): a qualitative study of patients' experiences of involvement in decision making. Physiotherapy 102(4), 339-344

Stilwell P and Harman K (2017) 'I didn't pay her to teach me how to fix my back': a focused ethnographic study exploring chiropractors' and chiropractic patients' experiences and beliefs regarding exercise adherence. Journal of the Canadian Chiropractic Association 61(3), 219-230

Stomski N J and Mackintosh S F; Stanley M (2014) The experience of acupuncture care from the perspective of people with chronic low back pain: a grounded theory study. Acupuncture in Medicine 32(4), 333-9

Toye F and Barker K (2012) 'I can't see any reason for stopping doing anything, but I might have to do it differently'--restoring hope to patients with persistent non-specific low back pain--a qualitative study. Disability & Rehabilitation 34(11), 894-903

Toye F and Barker K (2012) Persistent non-specific low back pain and patients' experience of general practice: a qualitative study. Primary Health Care Research & Development 13(1), 72-84

Valenzuela-Pascual F, Garcia-Martinez E and Molina-Luque F ; Soler-Gonzalez J ; Blanco-Blanco J ; Rubi-Carnacea F ; Climent-Sanz C ; Briones-Vozmediano E ; (2021) Patients' and primary healthcare professionals' perceptions regarding chronic low back pain and its management in Spain: a qualitative study. Disability & Rehabilitation 43(18), 2568-2577

White S and Seibold C (2008) Walk a mile in my shoes: an auto-ethnographic study. Contemporary Nurse: A Journal for the Australian Nursing Profession 30(1), 57-68

Zufferey M C and Schulz P J; (2013) [Learning self-management for pain. Potentials and limits of an Internet website for low back pain]. Revue Medicale Suisse 9(392), 1366-9

## Wrong topic of interest (n = 18)

Clarke Amanda, Anthony Geraldine and Gray Denise ; Jones Derek ; McNamee Paul ; Schofield Patricia ; Smith Blair H; Martin Denis ;. 2012. ""I feel so stupid because I can't give a proper answer…" How older adults describe chronic pain: a qualitative study.". *Bmc Geriatrics* 12(1):78-78.

Gillsjö Catharina, Schwartz-Barcott Donna and Bergh Ingrid ;. 2013. "Learning to Endure Long-Term Musculoskeletal Pain in Daily Life at Home: A Qualitative Interview Study of the Older Adult's Experience". *Journal Of Gerontology And Geriatric Research* 2(4):1-11.

Gonzalez A I, Ramtin S and Ring D ; Donthula D ; Queralt M ;. 2022. "People Have Mixed Reactions to Both Physiological and Psychological Explanations of Disproportionate Pain". *Clinical Orthopaedics & Related Research* 08:08.

Horment-Lara G, Luttges-Sciaccaluga C and Espinoza-Ordonez C ; Aliaga-Castillo V ;. 2022. ""I don't want to be a burden" A qualitative study of the beliefs of women with chronic low back pain in relation to their painful experience". *Musculoskeletal Science & Practice* 59:102539.

Iles R A, Taylor N F; Davidson M and O'Halloran P D;. 2012. "Patient recovery expectations in non-chronic non-specific low back pain: a qualitative investigation". *Journal of Rehabilitation Medicine* 44(9):781-7.

Katz J N, Lyons N and Wolff L S; Silverman J ; Emrani P ; Holt H L; Corbett K L; Escalante A ; Losina E ;. 2011. "Medical decision-making among Hispanics and non-Hispanic Whites with chronic back and knee pain: a qualitative study". *BMC Musculoskeletal Disorders* 12:78.

Kligler B, Buonora M and Gabison J ; Jacobs E ; Karasz A ; McKee M D;. 2015. ""I Felt Like It Was God's Hands Putting the Needles In": A Qualitative Analysis of the Experience of Acupuncture for Chronic Pain in a Low-Income, Ethnically Diverse, and Medically Underserved Patient Population". *Journal of Alternative & Complementary Medicine* 21(11):713-9.

Madill E S, Samuels R and Newman D P; Boudreaux-Kelley M ; Weiner D K;. 2019. "Development of an Evaluative, Educational, and Communication-Facilitating App for Older Adults with Chronic Low Back Pain: Patient Perceptions of Usability and Utility". *Pain Medicine* 20(11):2120-2128.

Nordstoga A L, Bach K and Sani S ; Wiratunga N ; Mork P J; Villumsen M ; Cooper K ;. 2020. "Usability and Acceptability of an App (SELFBACK) to Support Self-Management of Low Back Pain: Mixed Methods Study". *JMIR Rehabilitation And Assistive Technologies* 7(2):e18729.

Oien A M, Råheim M and Iversen S ; Steihaug S ;. 2009. "Self-perception as embodied knowledge -- changing processes for patients with chronic pain". *Advances in Physiotherapy* 11(3):121-129.

Schmidt E, Schopf A C and Farin E ;. 2017. "What is competent communication behaviour of patients in physician consultations? - Chronically-ill patients answer in focus groups". *Psychology Health & Medicine* 22(8):987-1000.

Schulz P J, Rubinelli S and Mariotti G ; Keller N ;. 2009. "Meeting the ranging of informational needs of chronic low back pain sufferers: conceptual design and rationale of the interactive website ONESELF". *Disability & Rehabilitation* 31(25):2118-24.

Snelgrove S and Liossi C . 2009. "An interpretative phenomenological analysis of living with chronic low back pain". *British Journal of Health Psychology* 14(Pt 4):735-49.

Stamm O, Dahms R and Muller-Werdan U ;. 2020. "Virtual reality in pain therapy: a requirements analysis for older adults with chronic back pain". *Journal of Neuroengineering & Rehabilitation* 17(1):129.

Stensland M L and Sanders S. 2018. "Not So Golden After All: The Complexities of Chronic Low Back Pain in Older Adulthood". *Gerontologist* 58(5):923-931.

Stensland M. 2021. ""If You Don't Keep Going, You're Gonna Die": Helplessness and Perseverance Among Older Adults Living With Chronic Low Back Pain". *Gerontologist* 61(6):907-916.

Strong Jenny, Ashton Roderick and Chant David ; Cramond Tess ;. 1994. "An Investigation of the Dimensions of Chronic Low Back Pain: the Patients' Perspectives". *British Journal of Occupational Therapy* 57(6):204-208.

Walker J, Sofaer B and Holloway I ;. 2006. "The experience of chronic back pain: accounts of loss in those seeking help from pain clinics". *European Journal of Pain* 10(3):199-207.

## Wrong study design (n = 7)

Cino Kathleen. 2014. "Aromatherapy Hand Massage for Older Adults With Chronic Pain Living in Long-Term Care:". *Journal Of Holistic Nursing* 32(4):304-313.

Combs M A and Thorn B E;. 2015. "Yoga attitudes in chronic low back pain: Roles of catastrophizing and fear of movement". *Complementary Therapies in Clinical Practice* 21(3):160-5.

Angel S. 2022. "Helpful factors in a healthcare professional intervention for low‐back pain: Unveiled by Heidegger's philosophy". *Nursing Philosophy* 23(1):1-11.

Iles R A, Taylor N F; Davidson M and O'Halloran P ;. 2014. "An effective coaching intervention for people with low recovery expectations and low back pain: a content analysis". *Journal of Back & Musculoskeletal Rehabilitation* 27(1):93-101.

Lane P. 2000. "Adults with chronic low back pain felt frustrated, unsupported, and powerless with healthcare, social, and legal systems [commentary on Walker J, Holloway I, Sofaer B. In the system: the lived experience of chronic back pain from the perspectives of those seeking help from pain clinics. PAIN 1999 Apr;80:621-8]". *Evidence Based Nursing* :29-29.

Lewis K, Metcalfe S and Pearson T ; Whichello R ;. 2019. "Implementing Yoga Into the Management of Patients With Refractory Low Back Pain in an Outpatient Clinic Setting". *Journal of Holistic Nursing* 37(3):238-247.

Stenner P, Cross V and McCrum C ; McGowan J ; Defever E ; Lloyd P ; Poole R ; Moore A P;. 2015. "Self-management of chronic low back pain: Four viewpoints from patients and healthcare providers". *Health Psychology Open* 2(2):2055102915615337.

## Exclude full text not available (n=2)

Cassidy E, Cooke G and Hurley D A;. 2008. "Patient outcomes and experiences of two exercise approaches for chronic low back pain...annual conference 'Radharc Eile -- A Different View': Radisson SAS, Galway, Friday 16th and Saturday 17th November 2007". *Physiotherapy Ireland* 29(1):71-71.

Eaves Emery, Ritenbaugh Cheryl and Hsu Clarissa ;. 2014. "Changing Hopes and Expectations of CAM Treatments for Back Pain: A Longitudinal Qualitative Analysis". *Journal of Alternative & Complementary Medicine* 20(5):A141-A141.
