# Supplementary material for: Acceptability, values, and preferences of older people for chronic low back pain management; a qualitative evidence synthesis
Source: BMC Geriatr. 2024 Jan 5;24:24. doi: 10.1186/s12877-023-04608-4 (PMC10768085; doi:10.1186/s12877-023-04608-4)
Supplement: Supplementary file 4 — Additional file 4. Evidence profile table. [file 12877_2023_4608_MOESM4_ESM.docx]

**Additional file 4: Evidence profile table**

| **#** | **Summarised review finding** | **Methodological limitations** | **Coherence** | **Adequacy** | **Relevance** | **GRADE-CERQual assessment of confidence** | **References** |
| --- | --- | --- | --- | --- | --- | --- | --- |
| **1- VALUES AND PREFERENCES** | | | | | | | |
| 1 | Many participants preferred providers who treated them with respect, cared for their individual needs and recognized the patient as the expert. They became frustrated when they were not taken seriously, disbelieved, were not treated as a person, experienced a lack of dialogue or clear, specific information or other conditions were prioritized over their CPLBP. This could be a deterrent to future treatment or care seeking. Many participants valued a health care provider who understood, listened, and remembered them. If this was lacking participants could feel not cared about, vulnerable, or alone with their pain. They wanted meaningful relationships with their providers who could sympathize, understand, and see them as a whole person. This understanding could help to legitimize and validate their pain experience. | Minor concerns  **Explanation:** Minor concerns regarding methodological limitations because of lack of reporting of reflexivity in some studies. | No/Very minor concerns | No/Very minor concerns | No/Very minor concerns | High confidence  **Explanation:** Minor concerns regarding methodological limitations, No/Very minor concerns regarding coherence, No/Very minor concerns regarding adequacy, and No/Very minor concerns regarding relevance | Allvin R et al. 2019; Dima A et al. 2013; Igwesi-Chidobe C N et al. 2017; Lin I B et al. 2013; Lin I et al. 2014; Lyons Kevin J et al. 2013; Makris Una E et al. 2015; Rodriguez I et al. 2019; Teh Carrie F et al. 2009; |
| 2 | Participants generally emphasized that there was a need for individualized care and guidance (for example how to perform an exercise) across the different interventions, whether health-professional or peer delivered. Care should be person-centred and provide continuity. Supervision/professional guidance allowed older people to feel safe. Several participants reported the importance of having an instructor for exercise or group classes who was personable, knowledgeable, and interactive and gave each participant individual attention. There was a preference for a collaborative communication style. | Minor concerns  **Explanation:** Minor concerns regarding methodological limitations because of lack of reflexiity in many studies and a finding that describes people's interactions with health care workers | Moderate concerns  **Explanation:** Moderate concerns regarding coherence because of a large amount of variation in the data and how each study contributes ot the overall finding. | Minor concerns  **Explanation:** Minor concerns regarding adequacy because of thin data from some studies. | Minor concerns  **Explanation:** Minor concerns regarding relevance because of a majorty of high income settings | Low confidence  **Explanation:** Minor concerns regarding methodological limitations, Moderate concerns regarding coherence, No/Very minor concerns regarding adequacy, and Minor concerns regarding relevance | Cooper K et al. 2017; Dima A et al. 2013; Hay M E & Connelly D M 2020; Igwesi-Chidobe C N et al. 2019; Igwesi-Chidobe C N et al. 2020; Kuss K et al. 2016; Lee T L et al. 2020; Lilje S C et al. 2017; Lin I et al. 2014; Lyons Kevin J et al. 2013; Teh Carrie F et al. 2009; |
| 3 | Participants generally agreed that there should be collaboration and communication across care teams/ different healthcare providers to ensure adequate treatment and to avoid duplication in testing, treatments and ensure consistency of recommendations and information across providers. Some participants had experienced no or a lack of communication between their health care providers (within or across specialties) concerning their diagnosis and plan of care or had received conflicting advice. | No/Very minor concerns | No/Very minor concerns | Minor concerns  **Explanation:** Minor concerns regarding adequacy because of thin data from one study. | Moderate concerns  **Explanation:** Moderate concerns regarding relevance because of limited settings | Moderate confidence  **Explanation:** No/Very minor concerns regarding methodological limitations, No/Very minor concerns regarding coherence, Minor concerns regarding adequacy, and Moderate concerns regarding relevance | Allvin R et al. 2019; Lin I et al. 2014; Lyons Kevin J et al. 2013; |
| 4 | It was important for participants to receive a diagnosis. This influenced their treatment decisions, self-management decisions and how they viewed themselves and their prognosis. The way a diagnosis is communicated can shape the patient's beliefs and response to their CPLBP. Inadequate or incorrect information influenced participants to view their diagnosis as threatening and as a reason for changing their daily activities. Participants preferred clear, honest and adequate information about diagnosis and prognosis and treatment. | Minor concerns  **Explanation:** Minor concerns regarding methodological limitations because of unclear methods reporting from some studies | Moderate concerns  **Explanation:** Moderate concerns regarding coherence because of varied data and opaque data from a few studies. | No/Very minor concerns | No/Very minor concerns | Moderate confidence  **Explanation:** Minor concerns regarding methodological limitations, Moderate concerns regarding coherence, No/Very minor concerns regarding adequacy, and No/Very minor concerns regarding relevance | Bonfim I D. S et al. 2021; Dima A et al. 2013; Igwesi-Chidobe C N et al. 2017; Igwesi-Chidobe C N et al. 2019; Kuss K et al. 2016; Lin I B et al. 2013; Lin I et al. 2014; Lyons Kevin J et al. 2013; MacKichan F et al. 2013; Rodriguez I et al. 2019; |
| 5 | Some participants expressed dissatisfaction with health care providers for being unwilling to discuss treatment options other than medication. Some of these participants had different priorities than their health care providers and felt that their providers were not meeting their needs by only providing medication and giving inadequate or conflicting information. | Minor concerns  **Explanation:** Minor concerns regarding methodological limitations because of lack of reporting of reflexivity. We feel the researchers position or background could have influenced how respondents answered the question. | Minor concerns  **Explanation:** Minor concerns regarding coherence because some slightly opaque data. | Moderate concerns  **Explanation:** Moderate concerns regarding adequacy because of somewhat thin data from two studies and very thin data from 1. | Moderate concerns  **Explanation:** Moderate concerns regarding relevance because only HIC settings and one study where all participants were not over 60. | Low confidence  **Explanation:** Minor concerns regarding methodological limitations, Minor concerns regarding coherence, Moderate concerns regarding adequacy, and Moderate concerns regarding relevance | Allvin R et al. 2019; Makris Una E et al. 2015; Teh Carrie F et al. 2009; |
| 6 | Patients had clear preferences and values for how they chose a specific treatment for CPLBP, which could be influenced by previous experiences. They valued and preferred treatments that they experienced as effective, beneficial and credible. In some cases, they also valued treatments that fit them as individuals (personally enjoyable, positive impact, meaningful, involved social engagement). | No/Very minor concerns | Minor concerns  **Explanation:** Minor concerns regarding coherence because of small variations related to type of intervention. Some focusing more on specific parts of the finding than others. | No/Very minor concerns | No/Very minor concerns | High confidence  **Explanation:** No/Very minor concerns regarding methodological limitations, Minor concerns regarding coherence, No/Very minor concerns regarding adequacy, and No/Very minor concerns regarding relevance | Dima A et al. 2013; Hay M E & Connelly D M 2020; Igwesi-Chidobe C N et al. 2019; Igwesi-Chidobe C N et al. 2020; Kirby E R et al. 2014; Kuss K et al. 2016; Lee T L et al. 2020; Leonhardt Corinna et al. 2017; Lilje S C et al. 2017; Makris Una E et al. 2015; Rodriguez I et al. 2019; Stensland M 2021; Teh Carrie F et al. 2009; |
| 7 | Many participants experienced that medication was often the only intervention that made a difference to the severity of their pain. However, they were apprehensive of, or dissatisfied with, medication for a number of reasons, often viewing it as a quick fix, temporary relief, or that it just masked the pain. Many participants were apprehensive of taking too many medications, the side effects, risk of addiction or did not like how the medications made them feel. Some avoided taking medication all together, filling prescriptions or adjusted medication themselves because of perceived risks of adverse events. | Minor concerns  **Explanation:** Minor concerns regarding methodological limitations because of lack of reporting around reflexivity. We feel that the researcher's position or role could have influenced how patients answered the question. | No/Very minor concerns | No/Very minor concerns | Moderate concerns  **Explanation:** Moderate concerns regarding relevance because all except one study from USA. 2 studies had some participants under 60. | Moderate confidence  **Explanation:** Minor concerns regarding methodological limitations, No/Very minor concerns regarding coherence, No/Very minor concerns regarding adequacy, and Moderate concerns regarding relevance | Cummings E C et al. 2017; Dima A et al. 2013; Lyons Kevin J et al. 2013; Makris Una E et al. 2015; Stensland M 2021; Teh Carrie F et al. 2009; |
| 8 | Many participants expressed a fear of addiction to medication, especially to opioid analgesics. This led them to not fill prescriptions, to adjust the dosage or stop taking the medication often without consulting their health care provider. In one case, the fear of addiction came from the health care provider who then refused to give the prescription requested. | Minor concerns  **Explanation:** Minor concerns regarding methodological limitations because of lack of reporting of reflexivity | No/Very minor concerns | Minor concerns  **Explanation:** Minor concerns regarding adequacy because some thinner data but speaking about a very specific topic | Moderate concerns  **Explanation:** Moderate concerns regarding relevance because all but one study from the USA and 1 study with some participants under 60. | Moderate confidence  **Explanation:** Minor concerns regarding methodological limitations, No/Very minor concerns regarding coherence, Minor concerns regarding adequacy, and Moderate concerns regarding relevance | Dima A et al. 2013; Lyons Kevin J et al. 2013; Makris Una E et al. 2015; Stensland M 2021; Teh Carrie F et al. 2009; |
| 9 | Mindfulness and meditation encouraged participants to examine, assess, understand and accept their pain rather than avoid it. It allowed some participants to increase their body awareness in relation to, for example, breathing, posture, cognition and pain, resulting in a perceived decrease in the significance or power of their pain experience. Others were able to use mindfulness and meditation for pain management and coping to varying degrees. | Minor concerns  **Explanation:** Minor concerns regarding methodological limitations because of unclear reporting of context, sampling and reflexivity. | No/Very minor concerns | No/Very minor concerns | Moderate concerns  **Explanation:** Moderate concerns regarding relevance because all studies took place in the USA and are part of a trial so the participants attendance to the interventionmay have been influenced. | Moderate confidence  **Explanation:** Minor concerns regarding methodological limitations, No/Very minor concerns regarding coherence, No/Very minor concerns regarding adequacy, and Moderate concerns regarding relevance | Lee T L et al. 2020; Luiggi-Hernandez J G et al. 2018; Morone N E et al. 2008; |
| 10 | Many participants liked a group format for physical exercise classes as these facilitated social support, collaborative learning and social activities which encouraged increased attendance. Participants in one study had a preference for shorter sessions on specific days to fit with their daily schedule. | Minor concerns  **Explanation:** Minor concerns regarding methodological limitations because of poor reporting in some studies on context, sampling and reflexivity. | Minor concerns  **Explanation:** Minor concerns regarding coherence because of two studies with data variation a little bit on the side. | Minor concerns  **Explanation:** Minor concerns regarding adequacy because of thin data from a few studies | Minor concerns  **Explanation:** Minor concerns regarding relevance because of some diversity in settings but missing others. | Moderate confidence  **Explanation:** Minor concerns regarding methodological limitations, Minor concerns regarding coherence, Minor concerns regarding adequacy, and Minor concerns regarding relevance | Dima A et al. 2013; Hay M E & Connelly D M 2020; Igwesi-Chidobe C N et al. 2019; Igwesi-Chidobe C N et al. 2020; Kuss K et al. 2016; Lee T L et al. 2020; |
| 11 | Participants broadly had positive views of peer support although they found it was difficult to access and did not know of support groups in their area. Empathy and "being believed" through common experience were the most important attributes in a peer supporter. Participants believed it would be helpful to share information and receive or exchange support and advice. | Moderate concerns  **Explanation:** Moderate concerns regarding methodological limitations because of lack of reporting of sampling, context and reflexivity from some studies. | Minor concerns  **Explanation:** Minor concerns regarding coherence because of the indirect nature of some of the data that needed to be interpreted. | Minor concerns  **Explanation:** Minor concerns regarding adequacy because of an ok amount of data from all studies. | Moderate concerns  **Explanation:** Moderate concerns regarding relevance because of teh studies being limited to HIC contexts and a number of studies where not all participants are over 60. | Low confidence  **Explanation:** Moderate concerns regarding methodological limitations, Minor concerns regarding coherence, Minor concerns regarding adequacy, and Moderate concerns regarding relevance | Cooper K et al. 2017; Cummings E C et al. 2017; Hay M E & Connelly D M 2020; MacKichan F et al. 2013; Teh Carrie F et al. 2009; |
| 12 | Participants wanted educational materials for physical interventions which had drawings and descriptions of the exercises. This made them more comprehensible, easier to follow and helpful for present and future reference. | Minor concerns  **Explanation:** Minor concerns regarding methodological limitations because of unclear reporting of sampling in a few studies and unclear reporting of reflexivity across all studies. | No/Very minor concerns | Serious concerns  **Explanation:** Serious concerns regarding adequacy because of thin data and a small number of studies. | Serious concerns  **Explanation:** Serious concerns regarding relevance because of studies from only two countries and one study where not all participants were over 60. | Low confidence  **Explanation:** Minor concerns regarding methodological limitations, No/Very minor concerns regarding coherence, Serious concerns regarding adequacy, and Serious concerns regarding relevance | Dima A et al. 2013; Igwesi-Chidobe C N et al. 2019; Igwesi-Chidobe C N et al. 2020; Kuss K et al. 2016; Leonhardt Corinna et al. 2017; |
| **2- COST/RESOURCES** | | | | | | | |
| 13 | Some participants viewed burden related to the intervention (financial, time and travel) as a barrier to accessing care. High cost rendered treatment inaccessible or deterred them from trying to adjust or continue with a recommended treatment. For others, who had the financial means or were accessing publicly funded health care, cost was not discussed. | No/Very minor concerns | No/Very minor concerns | Minor concerns  **Explanation:** Minor concerns regarding adequacy because of thin data from one study. | Minor concerns  **Explanation:** Minor concerns regarding relevance because of some participants under the age of 60. | Moderate confidence  **Explanation:** No/Very minor concerns regarding methodological limitations, No/Very minor concerns regarding coherence, Minor concerns regarding adequacy, and Minor concerns regarding relevance | Dima A et al. 2013; Igwesi-Chidobe C N et al. 2017; Lyons Kevin J et al. 2013; Stensland M 2021; |
| 14 | Many participants had a preference for health care providers that were in close proximity to where they lived. For some, this was due to their CLBP limiting their ability to travel more than short distances due to pain. If services were located a distance away, they were perceived as insufficient, inaccessible or that the distance was a barrier to care. However, some participants were willing to travel if a trusted or favored health care provider relocated or they were exploring new treatment options. Others preferred to find a new practitioner close to where they lived. | No/Very minor concerns | Minor concerns  **Explanation:** Minor concerns regarding coherence because of diverse specificity of findings across the studies | Minor concerns  **Explanation:** Minor concerns regarding adequacy because of thin data from some studies | Minor concerns  **Explanation:** Minor concerns regarding relevance because some studies had participants under 60 | Moderate confidence  **Explanation:** No/Very minor concerns regarding methodological limitations, Minor concerns regarding coherence, Minor concerns regarding adequacy, and Minor concerns regarding relevance | Allvin R et al. 2019; Dima A et al. 2013; Igwesi-Chidobe C N et al. 2017; Kirby E R et al. 2014; Lin I et al. 2014; Lyons Kevin J et al. 2013; Stensland M 2021; |
| **3- FEASIBILITY** | | | | | | | |
| 15 | Some participants found information about treatments difficult to access and assess on their own. They wanted help navigating the information they had found from a health or care provider or a peer support system in order to make a decision about treatment. | Minor concerns  **Explanation:** Minor concerns regarding methodological limitations because of unclear reporting of sampling in some studies and reflexivity in all studies. | No/Very minor concerns | Moderate concerns  **Explanation:** Moderate concerns regarding adequacy because of thin data in some studies. | Moderate concerns  **Explanation:** Moderate concerns regarding relevance because of all studies from high income countries and most studies where participants were not all over 60. | Low confidence  **Explanation:** Minor concerns regarding methodological limitations, No/Very minor concerns regarding coherence, Moderate concerns regarding adequacy, and Moderate concerns regarding relevance | Cummings E C et al. 2017; Dima A et al. 2013; Kirby E R et al. 2014; MacKichan F et al. 2013; Teh Carrie F et al. 2009; |
| 16 | Some participants adopted physical exercise, physical supports, or alternative forms of treatment (e.g., traditional or herbal medicine) as part of their self-management approach to supplement “conventional treatments” or when “conventional treatments” failed or were insufficient. This was often viewed as ‘experimenting’ to find a solution. Some participants did not inform their health care provider about these changes. | Minor concerns  **Explanation:** Minor concerns regarding methodological limitations because of unclear reporting of sampling and reflexivity. | Minor concerns  **Explanation:** Minor concerns regarding coherence because due to one negative case where the failure of alternative medicine pushed the participant to conventional medicine. | No/Very minor concerns | Minor concerns  **Explanation:** Minor concerns regarding relevance because of some studies where not all participants are over 60. | Moderate confidence  **Explanation:** Minor concerns regarding methodological limitations, No/Very minor concerns regarding coherence, No/Very minor concerns regarding adequacy, and Minor concerns regarding relevance | Cooper K et al. 2017; Hay M E & Connelly D M 2020; Igwesi-Chidobe C N et al. 2017; Igwesi-Chidobe C N et al. 2019; Lyons Kevin J et al. 2013; MacKichan F et al. 2013; Rodriguez I et al. 2019; Stensland M 2021; Teh Carrie F et al. 2009; |
| **4- EQUITY** | | | | | | | |
| 17 | Some participants felt that health care providers dismissed or minimized their CPLBP due to their age and often with ageist statements. They often felt that they were not taken seriously or "fobbed off", being told that pain was a natural consequence of ageing, and they should just "live with it". This could make them feel horrible or in some cases deter them from seeking further treatment. However, a few participants described being taken more seriously as they got older especially if they had an accompanying serious illness | Moderate concerns  **Explanation:** Moderate concerns regarding methodological limitations because of a lack of reporting around reflexivity and the finding is critical of health care providers so important to know who was doing teh researcher. Also unclear reporting of sampling in some studies. | No/Very minor concerns | Minor concerns  **Explanation:** Minor concerns regarding adequacy because of thin data from a few studies. | Moderate concerns  **Explanation:** Moderate concerns regarding relevance because of studies limited to high income countries and a number of studies where not all participants were over 60. | Low confidence  **Explanation:** Moderate concerns regarding methodological limitations, No/Very minor concerns regarding coherence, Minor concerns regarding adequacy, and Moderate concerns regarding relevance | Allvin R et al. 2019; Cooper K et al. 2017; Dima A et al. 2013; Hay M E & Connelly D M 2020; Lyons Kevin J et al. 2013; MacKichan F et al. 2013; Makris Una E et al. 2015; |
