# Supplementary material for: Acceptability, values, and preferences of older people for chronic low back pain management; a qualitative evidence synthesis
Source: BMC Geriatr. 2024 Jan 5;24:24. doi: 10.1186/s12877-023-04608-4 (PMC10768085; doi:10.1186/s12877-023-04608-4)
Supplement: Supplementary file 5 — Additional file 5. Final adapted framework. [file 12877_2023_4608_MOESM5_ESM.docx]

# Additional file 5: Final adapted framework

This framework is based in existing theory. The framework has been expanded to accommodate all the data relevant to our review question.

|  | | | Characteristics of the intervention (1) | | | Characteristics of the health care system (1) | | | | | Person centered care |
| --- | --- | --- | --- | --- | --- | --- | --- | --- | --- | --- | --- |
|  | | | Access, affordability and Availability (1, 2) | Feasibility of the intervention(1)  (time, distance, frequency of visits) | Stigma* | Involvement, Initiative and follow-up (2) | Communication and information (2) | Referral and care transitions (2) (Wait time, immediacy of treatment effect) | Coordination and cooperation (1, 2) (passive or active role in treatment) | Configuration of the care team (1) |  |
| PROGRESS Plus Framework (3) | Place of residence (Independent living or supported care) | HIC |  |  |  |  |  |  |  |  |  |
|  |  | LMIC |  |  |  |  |  |  |  |  |  |
|  | Race, ethnicity, culture, language | HIC |  |  |  |  |  |  |  |  |  |
|  |  | LMIC |  |  |  |  |  |  |  |  |  |
|  | Occupation (employed or retired) | HIC |  |  |  |  |  |  |  |  |  |
|  |  | LMIC |  |  |  |  |  |  |  |  |  |
|  | Gender, sex | HIC |  |  |  |  |  |  |  |  |  |
|  |  | LMIC |  |  |  |  |  |  |  |  |  |
|  | Religion | HIC |  |  |  |  |  |  |  |  |  |
|  |  | LMIC |  |  |  |  |  |  |  |  |  |
|  | Education | HIC |  |  |  |  |  |  |  |  |  |
|  |  | LMIC |  |  |  |  |  |  |  |  |  |
|  | Socioeconomic status | HIC |  |  |  |  |  |  |  |  |  |
|  |  | LMIC |  |  |  |  |  |  |  |  |  |
|  | Social capital | HIC |  |  |  |  |  |  |  |  |  |
|  |  | LMIC |  |  |  |  |  |  |  |  |  |

*From the commissioner

1. Chua J, Briggs AM, Hansen P, Chapple C, Abbott JH. Choosing interventions for hip or knee osteoarthritis-what matters to stakeholders? A mixed-methods study. Osteoarthritis and Cartilage Open. 2020;2(3):100062.

2. Lawless MT, Marshall A, Mittinty MM, Harvey G. What does integrated care mean from an older person’s perspective? A scoping review. BMJ open. 2020;10(1):e035157.

3. O'Neill J, Tabish H, Welch V, Petticrew M, Pottie K, Clarke M, et al. Applying an equity lens to interventions: using PROGRESS ensures consideration of socially stratifying factors to illuminate inequities in health. Journal of clinical epidemiology. 2014;67(1):56-64.
